# Accumulation Rate, Depuration Kinetics, and Tissue Distribution of Polychlorinated Dibenzo-*p*-Dioxins and Dibenzofurans (PCDD/Fs) in Suckler Ewes (*Ovis aries*)

**DOI:** 10.1021/acs.jafc.4c02626

**Published:** 2024-06-17

**Authors:** Sylvain Lerch, Raphaël Siegenthaler, Jorge Numata, Jan-Louis Moenning, Frigga Dohme-Meier, Markus Zennegg

**Affiliations:** † Ruminant Nutrition and Emissions, Agroscope, 1725 Posieux, Switzerland; ‡ Research Contracts Animals, Agroscope, 1725 Posieux, Switzerland; § Department Safety in the Food Chain, German Federal Institute for Risk Assessment (BfR), Max-Dohrn-Str. 8-10, 10589 Berlin, Germany; ∥ Laboratory for Advanced Analytical Technologies, Empa, Überlandstrasse 129, 8600 Dübendorf, Switzerland

**Keywords:** persistent organic pollutants, biotransfer factor, ruminant food safety, soil, adipose tissue, sheep milk, liver

## Abstract

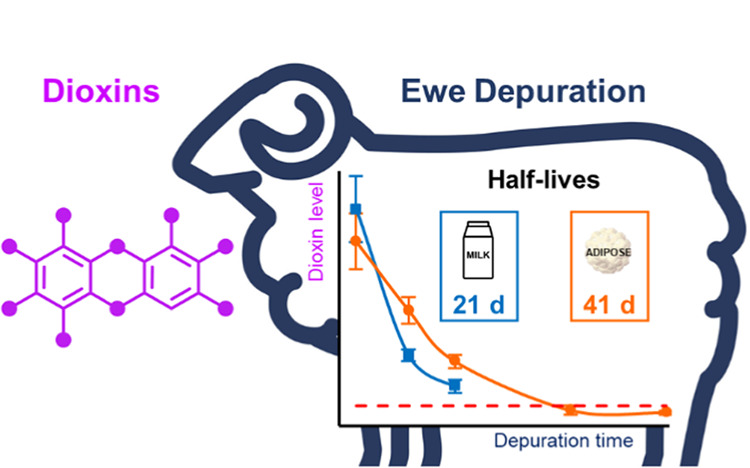

Understanding the transfer of polychlorinated dibenzo-*p*-dioxins and dibenzofurans (PCDD/Fs) in farm animals is essential for ensuring food safety, but such information for suckler ewes (*Ovis aries*) has been lacking. This work quantifies the accumulation, tissue distribution, and depuration kinetics of PCDD/Fs in these animals. Six suckler ewes (EXP group) were exposed to PCDD/Fs through contaminated hay (2.3–12.7 ng toxic-equivalent kg^–1^ dry matter) and then allowed to depurate by switching to noncontaminated hay from 29 days of lactation. Four control ewes were fed continuously with noncontaminated hay. At different time points covering depuration, weaning and slaughter, PCDD/F analysis of milk (three time points), blood and sternal adipose tissue (five time points), *Longissimus thoracis* muscle, liver, and empty body homogenate at slaughter (188 days of depuration) was performed. A relevant PCDD/F bioaccumulation was observed from oral intake in milk and adipose tissue (biotransfer factors of 1.24 and 1.06 day kg^–1^ lipids for the sum toxic-equivalent, respectively) in the EXP ewes, especially for penta- and hexa-chlorinated congeners. The EXP ewes’ adipose tissue started at 10-fold the EU maximum level (ML) and showed depuration below the ML after 130 days. Specific PCDD/F accumulation in the ewe liver was observed, especially for dibenzofurans. These toxicokinetic data can inform recommendations to ensure the chemical safety of sheep food products.

## Introduction

Polychlorinated dibenzo-*p*-dioxins and dibenzofurans (PCDD/Fs) are listed as persistent organic pollutants (POPs) in the Stockholm convention^1^ because of their multiple toxic end points (e.g., carcinogenicity, reprotoxicity, and endocrine disruption), environmental mobility, and persistence.^2^ The current consensus on the toxicological mode of action of PCDD/Fs involves the persistent activation of aryl hydrocarbon receptor signaling via the canonical pathway. Important adverse effects on physiological functions have been linked to it or suspected, such as immune, hepatic, cardiovascular, and reproductive systems disorders.^2^ These environmental contaminants are mostly formed and emitted through incomplete combustion processes of anthropogenic origin (unwanted formation during, e.g., industrial thermal processes and waste incinerations) and to a much lesser extent natural sources (i.e., volcanic activity, forest fires).^3^ After their dispersion and deposition on vegetation and soil, they persist long-term and may bioaccumulate in the animal food chain due to their lipophilicity and resistance to metabolism.^3,4^ Accordingly, humans are mainly exposed to these harmful compounds through the consumption of animal-derived foodstuffs (90% of the total human exposure),^2^ of which ruminant milk and meat account for 65%.^5^

Although successful measures have been taken since the 1980s to reduce anthropogenic PCDD/F emissions, environmental PCDD/F contamination (particularly of top-soil organic matter, which acts as a sink for POPs) is still detected sporadically in some areas.^3−5^ Recent incidents of soil contamination with PCDD/Fs include the “Land of Fires” in the Campania region of Italy due to illegal open burning of hazardous waste^6^ and a case in the Lausanne area of Switzerland due to an old municipal waste incinerator,^7^ which had a wide social and economic impact. In particular, livestock reared in such areas may be exposed orally to PCDD/Fs through ingestion of contaminated soil, such as herbivores grazing on pastures^8,9^ or poultry with access to open air runs.^10^ It can lead to food of animal origin (e.g., milk, meat, or eggs) exceeding the EU maximum level (ML, Regulation EU No 1259/2011), with subsequent destruction (i.e., confiscation and incineration) of herds and food products, threatening the sustainability of livestock production systems.^3−5^ Such episodic events not only endanger human health but also affect consumer confidence, disrupt the economy of the agrifood chain, and cause social hardship for farmers.

To ensure the safety of animal-derived foods, it is essential to quantify the transfer of PCDD/Fs from ingested feed and soil to animal products and estimate the time required to depurate formerly exposed herds. Risk assessors often rely on constant transfer factors or transfer rates derived from *in vivo* transfer experiments.^11,12^ For ruminant species, previous PCDD/Fs transfer studies have focused mostly on lactating cows,^13−17^ growing cattle,^15,18−20^ and lactating goats.^21,22^ Transfer studies describing the fate of PCDD/Fs in ovines are scarce, limited to one study in growing lambs under controlled conditions.^23^ Reports on lactating ewes are limited to a few field measurements of PCDD/F contents in milk, meat, and offal, with no measurements^24−26^ or only rough estimates^27^ of ewe PCDD/F exposure levels and the physiological traits that play a key role in the transfer of PCDD/Fs (i.e., milk fat yield and body fatness). Accordingly, the few available studies do not allow for calculation and interpretation of empirical transfer factors or depuration half-lives in adult sheep. Nevertheless, interspecies differences, particularly in PCDD/F tissue distribution and hepatic sequestration patterns, have been unraveled in cattle, sheep, and goats,^23−26^ further suggesting that accumulation and depuration rates differ between ruminant species. In addition, there is currently a growing global trend to use sheep flocks to maintain green spaces in urban and peri-urban areas (“ecopastoralism” practices).^28,29^ In such ruderalized environments, soil PCDD/Fs may reach moderate to high levels.^30^ Overall, this stresses the need to obtain toxicokinetic data on ewes for a better quantitative understanding of the PCDD/F transfer from feed and soil into sheep milk and meat. The aim of the present study was to gather toxicokinetic data in suckler ewes to quantify the accumulation (biotransfer factors, BTFs) and depuration (half-lives) in milk and adipose tissue as well as the tissue distribution of the regulated 2,3,7,8-chloro-substituted PCDD/Fs.

## Materials and Methods

### Animals and Diets

**Figure 1:**
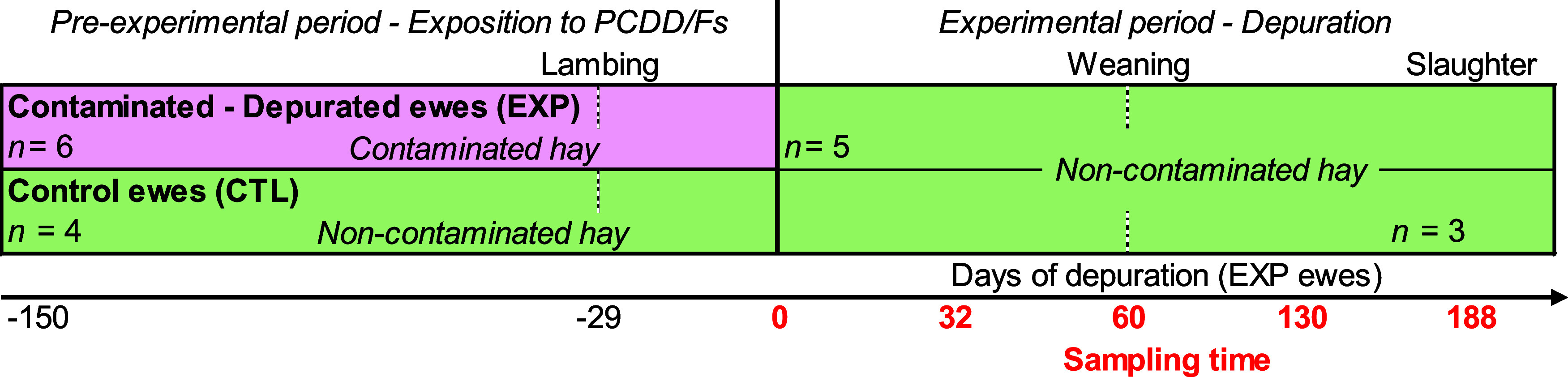
Summary of ewe polychlorinated dibenzo-*p*-dioxins and dibenzofurans (PCDD/Fs) exposure and depuration experimental design.

The experiment was approved with Number VD3750 by the Committee on Animal Experimentation of the canton Vaud, Switzerland, and took place from October 2021 to November 2022 on a farm in the Lausanne area (Switzerland, GPS decimal degree coordinates: 46.547310, 6.615216). Figure 1 summarizes the experimental design: ten multiparous gestating “Roux du Valais” ewes [*Ovis aries*, 53.9 ± 9.6 kg body weight (BW), 4.7 ± 1.6 years old on day 0 of the depuration experimental period] were recruited from the Lausanne sheep herd. The experimental (EXP) group (*n* = 6) originated from the most exposed individuals to PCDD/Fs (i.e., most often raised on pasture paddocks with the highest soil PCDD/F concentrations in Lausanne). From October 2021, EXP ewes were fed contaminated hay [PCDD/F concentration in the range 2.3–12.7 ng toxic-equivalent (TEQ) kg^–1^ dry matter (DM), Table 1] harvested in Lausanne. On 1 April 2022 (day 0 of the depuration experimental period), that is 29 ± 4 (mean ± SD) days after lambing, the EXP ewes were switched to noncontaminated hay (0.04 ng TEQ kg^–1^ DM, upper bound value, Table 1) harvested at Agroscope (Posieux, Switzerland, GPS decimal degree coordinates: 46.768824, 7.104856), which they received until the end of the experiment (day 188 of depuration). The control (CTL) group (*n* = 4) was composed of individuals that had mostly pastured on low to moderately contaminated areas of Lausanne over the past years and received noncontaminated hay (0.15 ng TEQ kg^–1^ DM, Table 1) from October 2021 until January 2022, and later on the noncontaminated hay from Agroscope. The ewes were fed hay ad libitum at 8:00 am as well as 350 g of DM day^–1^ per ewe concentrate (280 g after day 140 of depuration) and the same amount of pelleted whole maize (Table 1).

**Table 1:**
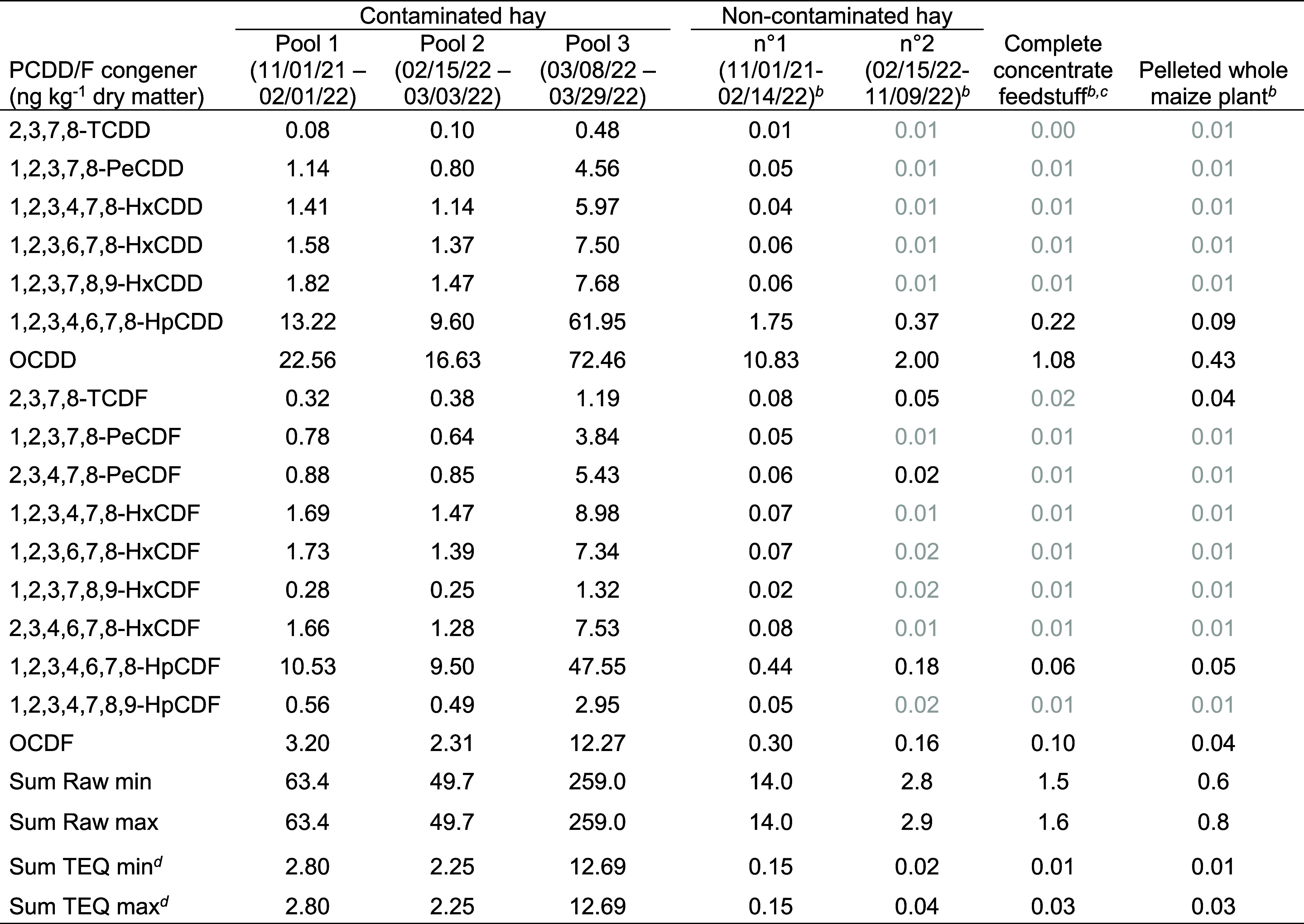
Feed Concentrations in Polychlorinated Dibenzo-*p*-Dioxins and Dibenzofurans (PCDD/Fs)^a^

a In gray, measurements below the limit of detection (LOD) are reported as equal to LOD.

b Mean of three successive pools.

c Sheep and goat organic feedstuff 16% crude protein, n°4785.2A, Anitech Moulin Chevalier SA, Cuarnens, Switzerland.

d Results normalized for TEQ are determined according to the WHO 2005 TEF values.^35^

The EXP and CTL groups were housed separately in two contiguous free-stall pens with hay refusals as bedding and had free access to water and a mineral salt block. Ewes were shorn twice during the study, 2–10 days before day 0 of the depuration period and before slaughter (day 188). Lambs were reared with their mothers from lambing until weaning, which occurred at 91 ± 4 days old (day 63 of depuration), and later ewes were nonlactating and nongestating. The ewes were slaughtered at the end of the experiment (day 188 of depuration, 6 October for EXP ewes and 10 November, 2022 for CTL ewes) at the experimental slaughterhouse of Agroscope Posieux according to legally defined procedures (stunning with a captive bolt followed by exsanguination).

Two ewes died unexpectedly during the study. One from the EXP group died on April 4, 2022, 3 days after the initiation of the depuration, following an acute lung viral infection. The second ewe from the CTL group died on August 14, 2022 (day 95 of the experimental period), presumably due to ruminal acidosis and liver steatosis caused by low hay intake due to dental infection. Both diagnoses were made following veterinary necropsies. Data from the EXP ewe that died were not included in the statistical analyses and are presented separately in Supporting Information, Tables S1 and S2, while data from the CTL ewe that died were retained for statistical analyses.

### Measurements, Sampling, and Analyses

#### Feed

Pools of each feedstuff composited over one to three month periods were ground for PCDD/F and nutrient analyses [DM, crude and acid insoluble ash, neutral and acidic detergent fibers, crude protein, ether extracted fat, starch (concentrate feed and pelleted whole maize plant only), and soil impurities; see Driesen et al.^15^ for the details of the analytical procedures and Supporting Information, Table S3].

#### Ewes

Ewe in vivo measurements and blood and sternal adipose tissue sampling were performed between 8:00 and 12:00 am (before feed distribution and after lamb separation from the ewes at 6:00 am) on depuration days 0 (start of the depuration period at a fixed date, 29 ± 4 days after lambing), 32 (61 ± 4 days after lambing, only for the EXP ewes), 60 (90 ± 4 days after lambing: time of weaning), 130 (only for EXP), and 188 (slaughter, Figure 1). No sample was taken prior to day 0 of depuration. The CTL ewes lambed on average 35 days after the EXP ewes, and the measurement and sampling schedules were adjusted accordingly. On each experimental day, ewes were weighed and scored for fat cover and conformation by visual and palpation grading (CH-TAX classification),^31^ and backfat thickness was measured by ultrasound between the fourth and fifth lumbar vertebrae^32^ using the equipment and procedures described by Xavier et al.^33^ Milk was sampled by hand milking (days 0, 32, and 60 only), after intramuscular injection of oxytocin whenever required (10–20 UI, Oxytobel, Bimeda, Dardilly, France), and stored at −20 °C until PCDD/F analysis. Blood was sampled by venipuncture at the jugular vein onto SiO_2_-coated tubes (Greiner Bio-One VACUETTE, Kremsmünster, Austria) with 9 mL for β-hydroxybutyrate and lipid class measurements (see Driesen et al.^15^ for details) and 45 mL for PCDD/F analyses. Additional blood (600 mL) was collected at exsanguination on day 188 (slaughter) for PCDD/F analysis. After clotting for 1 h at room temperature, the blood serum was separated via centrifugation (2400*g*, 15 min, ambient temperature) and stored at −20 °C until analyses. Subcutaneous sternal adipose tissue was harvested by biopsy, except on day 188, when it was collected post-mortem. The biopsy was performed through a 3 cm skin incision under local anesthesia (5 mL of 2% lidocaine, Vetoquinol AG, Bern, Switzerland) after the intramuscular administration of xylazine (0.5 mL of 100 kg^–1^ BW of 2% Rompun, Bayer HealthCare, Basel, Switzerland) and meloxicam (2.5 mL per 100 kg BW of 20 mg mL^–1^ Contacerra, Zoetis, Delémont, Switzerland). An adipose tissue sample of 20–100 mg was kept in Ringer’s solution at 39 °C for less than two hours before fixation in osmium acid for at least 10 days for subsequent adipose cell size measurement under a microscope.^15,33^ The remaining harvested adipose tissue (1–2 g by biopsy, >10 g at slaughter) was stored at −20 °C until PCDD/F analysis.

At slaughter (day 188), after exsanguination, the lower legs and head were removed, the carcass was dehided, and visceral tissues and organs were removed (digestive tract, bladder, liver, spleen, kidney, lung and trachea, heart, and omental, mesenteric, perirenal and pericardial adipose tissues). The digestive tract and bladder were emptied, and the digestive contents and urine weights were calculated by weight differences. Exsanguinated blood, hot and cold (4 °C for 24 h) carcasses, and every tissue and organ weights were recorded. In addition to blood and sternal adipose tissue, samples of liver (400–500 g) and *Longissimus thoracis* (LT) muscle (600–900 g, between the fifth and twelfth ribs) were cut into small pieces and stored at −20 °C until chemical composition (DM, lipids, proteins, and ashes; see Driesen et al.^34^ for details) and PCDD/F analyses were performed. The rest of the empty body (full body without digestive contents, urine, exsanguinated blood, adipose tissue, liver, and muscle samples) was frozen at −20 °C in a plastic box before being ground and homogenized thoroughly using four successive steps and devices (see Xavier et al.^33^ for details). Aliquots (900–1100 g) of empty body homogenate were stored at −20 °C until chemical composition and PCDD/F analyses. Such procedures allowed the body chemical composition and PCDD/F burdens to be determined. No sampling or measurement (except BW) was performed on the lambs.

### PCDD/F Analysis

#### Chemicals and Instruments

All of the organic solvents (Biosolve Chimie, Dieuze, France) were of pestanal grade. ^13^C_12_-labeled PCDD/F internal standards were purchased from Wellington Laboratories Inc. (Guelph, Canada). The EZprep 123 system from Fluid Management Systems (FMS, Billerica, MA) with an Extra High-Capacity Kit (EHCLS-KT05.0G, FMS) was used for sample cleanup and fractionation. For PCDD/F quantification, we used an atmospheric pressure gas chromatography-mass spectrometer (APGC-MS) with an Agilent 7693A autosampler and an Agilent 7890B GC, coupled to a Xevo TQ-XS triple quadrupole MS instrument (Waters, Milford, MA) and equipped with a Rtx-Dioxin2 capillary column (30 m length, 0.25 mm diameter, 0.25 μm film thickness; Restek Corporation, Bellefonte, PA).

#### Quality Assurance

All feed and ewe tissue samples for PCDD/F analyses were collected on either precleaned glassware (RBS-50 bath, dishwasher, heated at 450 °C, and rinsed with pestanal grade solvents) or aluminum plates. Several precautions and cleaning procedures with detergents and solvents were consistently applied throughout the sample preparation and analytical flow to mitigate any cross-contamination. To ensure the quality of PCDD/F quantification, procedural blanks were also performed for each sample type (Supporting Information, Table S4). The limit of detection (LOD) was calculated as five times the signal-to-noise ratio.

#### Sample Extraction, Cleanup, and APGC-MS Analysis of PCDD/Fs

Undried (feed, milk, blood serum, adipose tissue, and empty body homogenate) or dried (liver and muscle, 50 °C, 72 h, ground using a knife mill, Grindomix GM200, Retsch, Haan, Germany) samples were analyzed for the concentrations of the 17 2,3,7,8-chloro-substituted PCDD/F congeners as described by Driesen et al.^15,34^ Briefly, lipids were extracted by Soxhlet (solid samples) or liquid–liquid extraction (for defrozen milk and blood serum), prior to addition of ^13^C_12_-labeled internal standards and cleanup on acidic and basic silica, alumina, and activated carbon (EZprep 123, FMS). Quantification was further performed by APGC-MS in the daughter ion scan (MS-MS) using the isotope dilution technique. For kinetic blood samples on days 0, 32, 60, and 130, the amount of lipid extracted from individual ewes was low (<100 mg) and resulted in PCDD/F quantification below the LOD. Pooling of blood-extracted lipids from the four (CTL) or five (EXP) ewes within each sampling day (except at slaughter, day 188, where individual measurements were recorded) was further performed, allowing levels higher than LOD values to be recorded. The results normalized to TEQ were determined according to the World Health Organization 2005 toxic-equivalent factors,^35^ as those are currently in use in the European feed and food regulations (Regulations EU No 277/2012 and 1259/2011).

### Calculations and Statistical Analyses

Estimates of hay intake were made using the French feeding system for sheep from the Institut National de la Recherche Agronomique (INRA)^36^ with BW, body fatness score, lactation stage, lamb growing rate, ambient temperature, hay fill unit (from ash, crude protein, and neutral-detergent fiber contents using Prev’Alim software),^37^ and concentrate intake as predictive variates. Empty body lipid mass (and PCDD/F burdens) was determined at slaughter from the sum of lipid (or PCDD/F congener mass) in exsanguinated blood, sternal adipose tissue, liver, and LT muscle samples and in the rest of the empty body homogenate. Multiple linear regression was then performed using the GLM procedure of SAS (version 9.4, SAS Institute, Cary, NC) to relate empty body lipid mass to slaughter BW and body fatness score, according to eq 1:
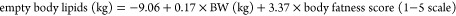
1*R*^2^ = 0.88, RMSE = 2.00 kg, residual coefficient of variation (rCV) = 19.2%, *n* = 10.

Equation 1 was further used to estimate the empty body lipid mass at each time point (days 0, 32, 60, and 130), given the measured BW and body fatness score.

*In vivo* kinetic data for measured physiological traits and PCDD/F concentrations in milk and sternal adipose tissue, as well as the post-mortem tissue PCDD/F concentrations and body composition and PCDD/F burdens, were analyzed using the MIXED procedure of SAS. The statistical model for kinetic repeated measures considers the measurement day (0, 32, 60, 130, and 188), treatment (EXP vs CTL), and the day × treatment interaction as fixed effects, and ewe as a random effect using a spatial power covariance structure. The statistical model for post-mortem tissue PCDD/F concentrations repeated measures included tissue (empty body, adipose tissue, LT muscle, liver, and blood), treatment, and the tissue × treatment interaction as fixed effects and ewe as a random effect using a first-order autoregressive covariance structure. The statistical model for post-mortem body composition and PCDD/F burdens included treatment as a fixed effect and ewe as a random effect using a variance component covariance structure. Logarithmic transformation was applied when needed to comply with the assumptions of normality, homoscedasticity, and linearity of residuals. When transformation was required, least-squares means and standard errors were reported from untransformed data, whereas *p*-values reflected transformed statistical analyses. Significance was declared at *p* ≤ 0.05 and trends considered at 0.05 < *p* ≤ 0.10.

To assess the accumulation and depuration rates, BTFs and depuration half-lives were computed, respectively. The BTFs from oral intake to milk and sternal adipose tissue were computed in the EXP ewes using day 0 (end of accumulation) PCDD/F measured concentrations and estimated oral intake, as shown in eq 2:
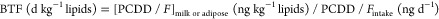
2

The BTFs were computed for 15 out of 17 congeners. Notably, 2,3,7,8-TCDF and 1,2,3,7,8,9-HxCDF were almost never detected in the milk and adipose tissue samples (<LOD). For the reliability of the BTF calculation, the assumption is made that steady state has been reached for milk and adipose tissue PCDD/F concentrations (i.e., the concentrations have reached a plateau following constant exposure) for every congener at day 0 in the EXP ewes. Indeed, in previous toxicokinetic investigations, steady state following constant exposure to PCDD/Fs was achieved after 6–153 days in milk of cows^13,14^ or goats,^22^ which is 1.2- to 30-fold shorter than the 180 days constant exposure of the EXP ewes to the contaminated hay in the present study.

For the half-life computations, monoexponential depuration models were fitted to the least-squares means of PCDD/F concentrations in milk and sternal adipose tissue of the EXP ewes, separately, using the NLIN procedure of SAS, according to eq 3:
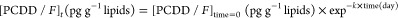
3

For adipose tissue, the concentration on day 188 was excluded, as it was not different (*p* > 0.10) from day 130 and led to worse fits (lower *R*^2^ and higher RMSE). The depuration half-life was calculated as shown in eq 4:
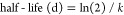
4

The depuration half-life was computed for 12 out of 17 congeners. 2,3,7,8-TCDF and 1,2,3,7,8,9-HxCDF measurements were lower than the LOD, and no satisfactory monoexponential decay could be fitted for 1,2,3,7,8-PeCDF, 1,2,3,4,7,8,9-HpCDF, or OCDF (too flat depuration curve). To test for statistical differences between half-lives in milk and adipose tissue for each congener, a *t*-test was used and confidence intervals for the half-lives were estimated. For both procedures, an estimate of the variance of the parameter for slope *k* was required and obtained using the delete-one jackknife method (as detailed in Supporting Information, Section S1).

## Results

### Feed PCDD/F Concentrations

The PCDD/F concentrations in hay, concentrate, and pelleted maize are listed in Table 1. Contamination levels were approximately 3-fold higher than those of the EU and Swiss ML (0.85 ng TEQ kg^–1^ DM, EU 277/2012 regulation) in the two first pools of contaminated hay fed to the EXP ewes from October 2021 to the beginning of March 2022. The third pool of hay fed to the ewes in March 2022 was remarkably 5-fold higher in PCDD/F level than the first two pools, that is, 15-fold the ML. Much lower PCDD/F concentrations were found in noncontaminated hay, concentrate, and pelleted maize (mean of the upper bounds of 0.06 ng TEQ kg^–1^ DM). For every feedstuff, 1,2,3,7,8-PeCDD was the most abundant congener in the TEQ sum (25–41%), followed by 2,3,4,7,8-PeCDF (9–15%). Dioxin-like polychlorinated biphenyls (PCBs) were analyzed in the contaminated hay, but their contribution to the total sum TEQ (PCDD/Fs + PCBs) was only of 14, 13, and 4% in the contaminated hay pools n°1, n°2, and n°3, respectively (data not shown). Accordingly, the fate of dioxin-like PCBs in suckler ewes was not investigated further.

### Ewe Physiological Traits

**Figure 2:**
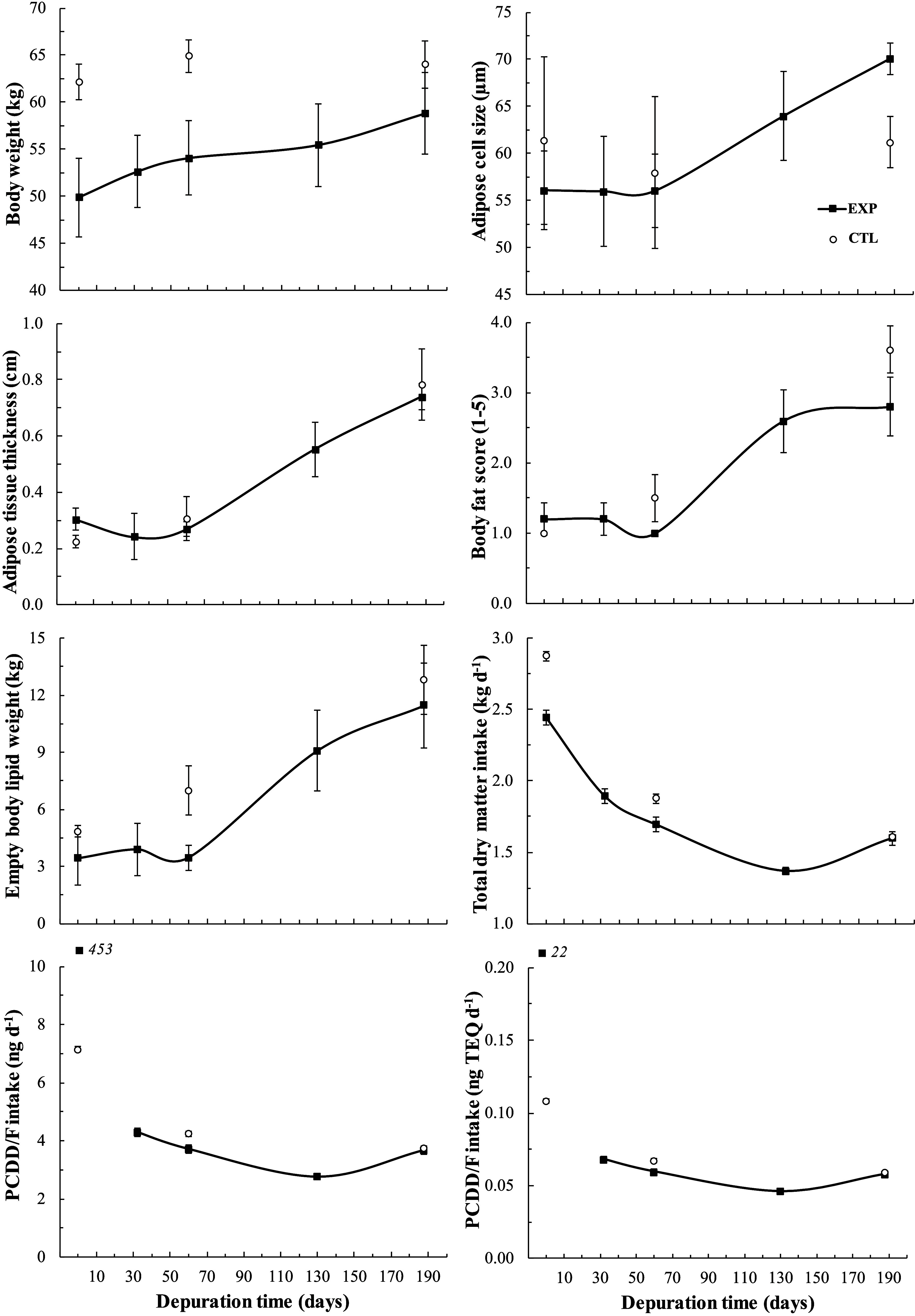
Kinetics in body weight and fatness, and intakes of dry matter and sum TEQ of polychlorinated dibenzo-*p*-dioxin and dibenzofurans (PCDD/Fs) in depurated (EXP, *n* = 5) and control (CTL, *n* = 4) ewes. Ewes were lactating until depuration day 63 (weaning time), and thereafter nonlactating and nongestating.

The BW, body fatness indicators, and intake estimates are shown in Figure 2 and Supporting Information, Table S5, and blood metabolites are shown in Supporting Information, Table S6. The BW increased steadily throughout the study in the EXP ewes but only during lactation (from day 0 to 60) in the CTL ewes. Accordingly, at depuration days 0 and 60, the BW was higher (*p* < 0.05) in the CTL than in the EXP ewes, whereas this difference disappeared at slaughter (day 188, *p* = 0.30). Body fatness indicators and body lipid mass estimates were low and did not change along lactation (days 0 to 60, *p* > 0.10) in either EXP or CTL ewes with similar values (*p* > 0.10) between treatments (average 4.7 kg of empty body lipids, Figure 2 and Supporting Information Table S5). Those traits increased (*p* < 0.01) sharply after weaning (days 60 to 188), reaching 12.2 kg of empty body lipids (average of the EXP and CTL ewes) at slaughter. Estimates of DM intake decreased over the depuration period in both the EXP and CTL ewes from 2.7 kg on day 0 to 1.6 kg of DM day^–1^ on day 188. The PCDD/F intake was much higher in the EXP ewes at the end of the exposure period (at day 0, 22 ng TEQ day^–1^) than during the depuration period and in the CTL ewes (average of 0.07 ng TEQ day^–1^, Figure 2). Regardless of the treatment, blood serum lipid classes (expect triglycerides) and β-hydroxybutyrate contents decreased (*p* < 0.01) from day 0 to day 188 of the experiment. In addition, the treatment effect was limited to a higher (*p* < 0.01) serum cholesteryl-ester content on day 0 in the CTL than in the EXP ewes (Supporting Information, Table S6).

### Kinetics in Milk, Blood, and Adipose Tissue PCDD/F Concentrations

**Figure 3:**
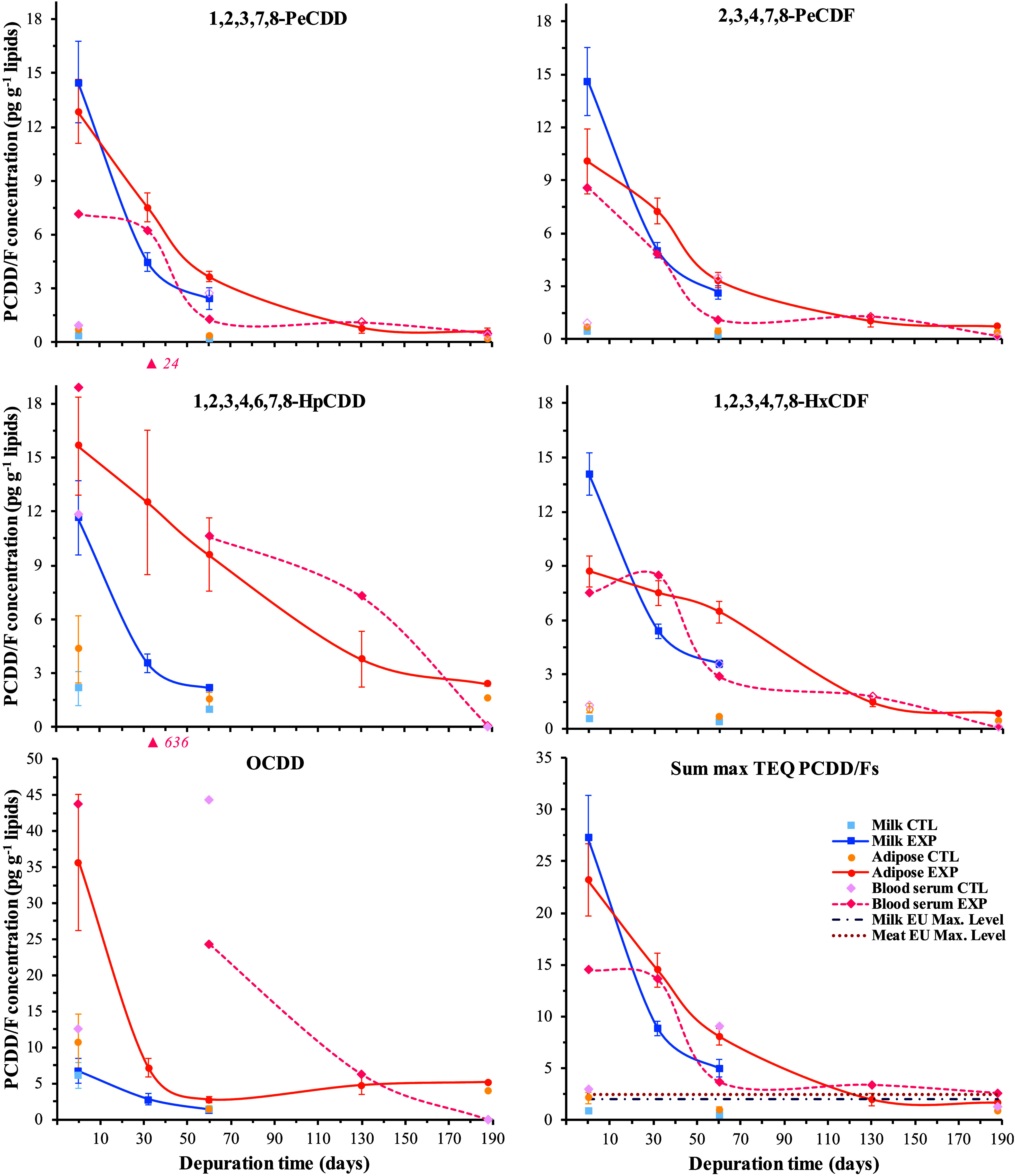
Depuration kinetics in milk, sternal subcutaneous adipose tissue, and blood serum concentrations of polychlorinated dibenzo-*p*-dioxins and dibenzofurans (PCDD/Fs) in depurated (EXP, *n* = 5) and control (CTL, *n* = 4) ewes. Empty symbols indicate measurements lower than the limit of detection. Ewes were lactating until depuration day 63 (weaning time), and thereafter nonlactating and nongestating.

**Figure 4:**
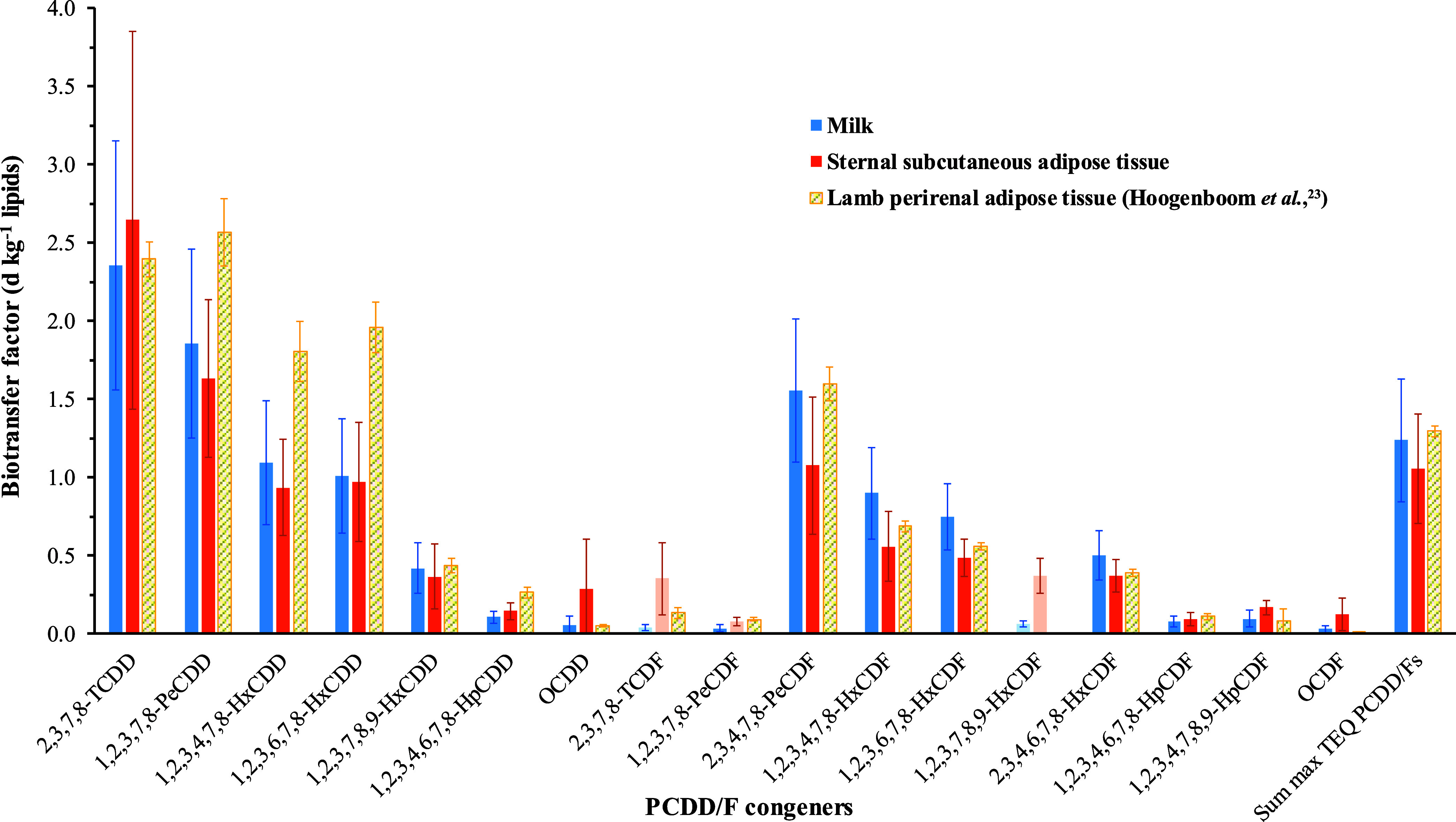
Feed to milk and sternal subcutaneous adipose tissue biotransfer factors of polychlorinated dibenzo-*p*-dioxins and dibenzofurans (PCDD/Fs) in ewes (present study, EXP treatment at the depuration day 0, *n* = 5) compared to lambs (Hoogenboom et al.,^23^ at 112 days of exposure, *n* = 4). Histograms in light color indicate measurements lower than the limit of detection.

Milk, blood, and adipose tissue kinetics for five illustrative PCDD/F congeners and in the sum TEQ are shown in Figure 3. Detailed results are available in Supporting Information, Tables S7–S9. Milk and adipose tissue BTFs determined on day 0 (end of exposure period) for EXP ewes are additionally reported in Figure 4, and monoexponential depuration half-lives in Table 2. Although the pooling strategy allowed more congeners to be detected, blood results should be interpreted with caution (Supporting Information, Table S8). Overall, PCDD/F levels recorded in blood serum over the depuration period, as well as the congener pattern, were in broad agreement with those in milk and adipose tissue.

**Table 2:** Monoexponential Depuration Half-Lives of Polychlorinated Dibenzo-*p*-dioxins and Dibenzofurans (PCDD/Fs) in Milk and Adipose Tissue of Depurated Suckler Ewes (EXP treatment of the present study, *n* = 5) Compared to Depurated Growing Lambs (Hoogenboom et al.,^23^*n* = 4) and Lactating Cows (Driesen et al.,^15^*n* = 2 Multiparous Cows; Lorenzi et al.,^14^*n* = 3)^a^^,^^b^

	milk^c^			adipose tissue^d^		
experiment	present study^e^	Driesen^15^	Lorenzi^14^	present study^e^	Hoogenboom^23^	Driesen^15^
animal	suckler ewes	low-yielding lactating cows	high-yielding lactating cows	suckler ewes	growing lambs	low-yielding lactating cows
depuration time	0, 32, 60 days	0, 35, 63 days	0, 28, 42 days	0, 32, 60, 130 days	0, 57 days	0, 124 days
2,3,7,8-TCDD	20 (15–32)^†^	78		31 (21–51)^†^	24	75
1,2,3,7,8-PeCDD	20 (15–30)*	56	13	36 (29–46)*	52	105
1,2,3,4,7,8-HxCDD	25 (20–35)*	39	12	57 (42–89)*	103	170
1,2,3,6,7,8-HxCDD	27 (21–40)*	55	18	52 (34–107)*	87	97
1,2,3,7,8,9-HxCDD	15 (12–20)*	42	16	32 (22–55)*	54	89
1,2,3,4,6,7,8-HpCDD	21 (17–26)**	30	51	72 (56–102)**	83	167
OCDD	26 (19–44)			14 (7–825)		
2,3,4,7,8-PeCDF	22 (18–28)**	48	21	43 (35–58)**	48	104
1,2,3,4,7,8-HxCDF	26 (17–51)^†^	32	19	74 (59–97)^†^	54	128
1,2,3,6,7,8-HxCDF	23 (19–29)**	38	14	55 (50–63)**	53	97
2,3,4,6,7,8-HxCDF	23 (20–25)**	31	15	48 (40–61)**	79	114
1,2,3,4,6,7,8-HpCDF	35 (29–45)**	20	29	87 (71–109)**	86	133
sum TEQ max^f^	21 (17–30)**	41	14	41 (33–52)**	39	110

a *, **, † Within row and for the present study in suckler ewes only, milk and adipose monoexponential depuration half-lives differed at *p* < 0.05, *p* < 0.01, or tended to differ at *p* < 0.10, respectively (*t*-test of the slopes of the monoexponential decay in milk vs adipose tissue for each congener using the jackknife method).

b The five depurated ewes (EXP) were formerly (until day 0) fed with a hay contaminated with PCDD/Fs, and further received a noncontaminated hay until 188 days of depuration. At day 0, ewes were lactating on average at 29 days in milk, weaning/dry-off took place at depuration day 63, and thereafter ewes were nonlactating and nongestating until the end of the depuration (day 188).

c Monoexponential depuration half-life was established based on three kinetic measurements.

d Monoexponential depuration half-life was established based on two (Hoogenboom et al.,^23^ and Driesen et al.,^15^) to four (present study) kinetic measurements.

e Monoexponential depuration half-life in the present study is for the kinetic from the means of the five EXP ewe’s measurements, with additionally reported in brackets the 95% confidence interval using the jackknife method.

f Results normalized for TEQ are determined according to the WHO 2005 TEF values.^35^

#### Congener Pattern

The congeners 1,2,3,7,8-PeCDD, 2,3,4,7,8-PeCDF, and 1,2,3,4,7,8-HxCDF were among the most abundant in the sum TEQ in the milk and adipose tissue of the EXP (on average 47%, 15%, and 6%, respectively) and CTL ewes (on average 35%, 13%, and 6%, respectively) and are representative of penta- and hexa-chlorinated congeners. The 1,2,3,4,6,7,8-HpCDD and OCDD are shown as representative of highly chlorinated congeners (Figure 3) and the most abundant congeners in the total PCDD/F raw concentrations (13 and 15% for 1,2,3,4,6,7,8-HpCDD and 13 and 29% for OCDD in the EXP and CTL ewes, respectively). Nonetheless, their contributions to the sum TEQ were negligible (1.0 and 2.1% for 1,2,3,4,7,8-HxCDF and 0.0 and 0.1% for OCDD in the EXP and CTL ewes, respectively). The 2,3,7,8-TCDF, 1,2,3,7,8-PeCDF, and 1,2,3,7,8,9-HxCDF were almost never detected, whereas 1,2,3,4,7,8,9-HpCDF and OCDF were detected mostly in the milk and adipose tissue of the EXP ewes on day 0 (Supporting Information, Tables S7 and S9).

#### Accumulation Rates

At the end of the exposure period (depuration day 0), the highest occurrence was found in the EXP ewes, with a slightly higher PCDD/F concentration in milk than in adipose tissue (27.3 vs 23.2 pg TEQ g^–1^ lipids, Figure 3). Those concentrations were 14- and 9-fold higher than the ML (2.0 and 2.5 pg TEQ g^–1^ lipids, in sheep milk and meat, respectively, EU 1259/2011 regulation), as well as 30- and 11-fold higher than in the CTL ewes (0.9 and 2.2 ng g^–1^ lipids, respectively, *p* < 0.001). Accordingly, the milk BTF for the sum TEQ on day 0 was numerically slightly higher (1.24 day kg^–1^ lipids) than in adipose tissue (1.06 day kg^–1^ lipids, Figure 4). A decrease (*p* < 0.01) in BTF was observed with increasing chlorination degree, from highest values for 2,3,7,8-TCDD, 1,2,3,7,8-PeCDD, and 2,3,4,7,8-PeCDF (average 1.92 day kg^–1^ lipids in milk and 1.78 day kg^–1^ lipids in adipose tissue) to lowest values for the three hepta- and two octa-chlorinated congeners (0.07 day kg^–1^ lipids in milk and 0.17 day kg^–1^ lipids in adipose tissue). The BTFs of the six detected hexa-chlorinated congeners fell in between (0.78 and 0.62 day kg^–1^ lipids, Figure 4). The only exceptions were 2,3,7,8-TCDF, 1,2,3,7,8-PeCDF, and 1,2,3,7,8,9-HxCDF, which were not detected in milk (except 1,2,3,7,8-PeCDF: BTF of 0.04 day kg^–1^ lipids) or adipose tissue (Supporting Information, Tables S7 and S9).

#### Depuration Kinetics

The exponential decays for the two penta-congeners and the sum TEQ in the EXP ewes along the depuration period showed curves with similar slopes, with numerically slightly higher slopes in milk than in adipose tissue (Figure 3). At the end of lactation (day 60), this led to 5.5- and 2.9-fold decreases compared to day 0 (*p* < 0.01) for the sum TEQ in milk and adipose tissue, respectively. Accordingly, higher concentrations were recorded in adipose tissue (8.1 pg TEQ g^–1^ lipids) than in milk (5.0 pg TEQ g^–1^ lipids) at the end of lactation. After weaning, adipose tissue concentrations continued decreasing (*p* < 0.001) at a comparable rate (4.1-fold decrease in 70 days), reaching 2.0 pg TEQ g^–1^ lipids on day 130, a level compliant with the ML in meat. The concentration did not change significantly further (*p* = 0.44) until day 188 (1.6 pg TEQ g^–1^ lipids), a level that was still higher than in the CTL ewes (0.8 pg TEQ g^–1^ lipids, *p* = 0.02). When compared to penta-chlorinated congeners, a similar exponential decay characterized the depuration of 1,2,3,4,6,7,8-HpCDD and 1,2,3,4,7,8-HxCDF in milk, whereas their adipose tissue concentrations decreased more slowly and in a linear fashion. Conversely, the adipose tissue OCDD concentration was higher on day 0 but decreased much faster over the first 30 days of depuration than in milk (Figure 3). Accordingly, shorter monoexponential half-lives (*p* < 0.05 for nine congeners) were consistently recorded in milk than in adipose tissue (2.3-fold difference on average, *n* = 11 congeners), with the exception of OCDD, for which a longer half-life was recorded in milk (26 days) than in adipose tissue (14 days, Table 2). Therefore, for the sum TEQ, the half-life was also shorter (*p* < 0.01) in milk (21 days) than in adipose tissue (41 days). In contrast to BTFs, no clear correlation between half-lives and PCDD/F chlorination degree was observed. The longest half-lives were observed for 1,2,3,4,6,7,8-HpCDF (on average, 61 days for milk and adipose tissue), followed by 1,2,3,4,7,8-HxCDF (50 days) and 1,2,3,4,6,7,8-HpCDD (47 days). The shortest were observed for 1,2,3,7,8,9-HxCDD (24 days), 2,3,7,8-TCDD (26 days), and 1,2,3,7,8-PeCDD (28 days, Table 2).

### PCDD/F Tissue Distribution and Body Burden

**Figure 5:**
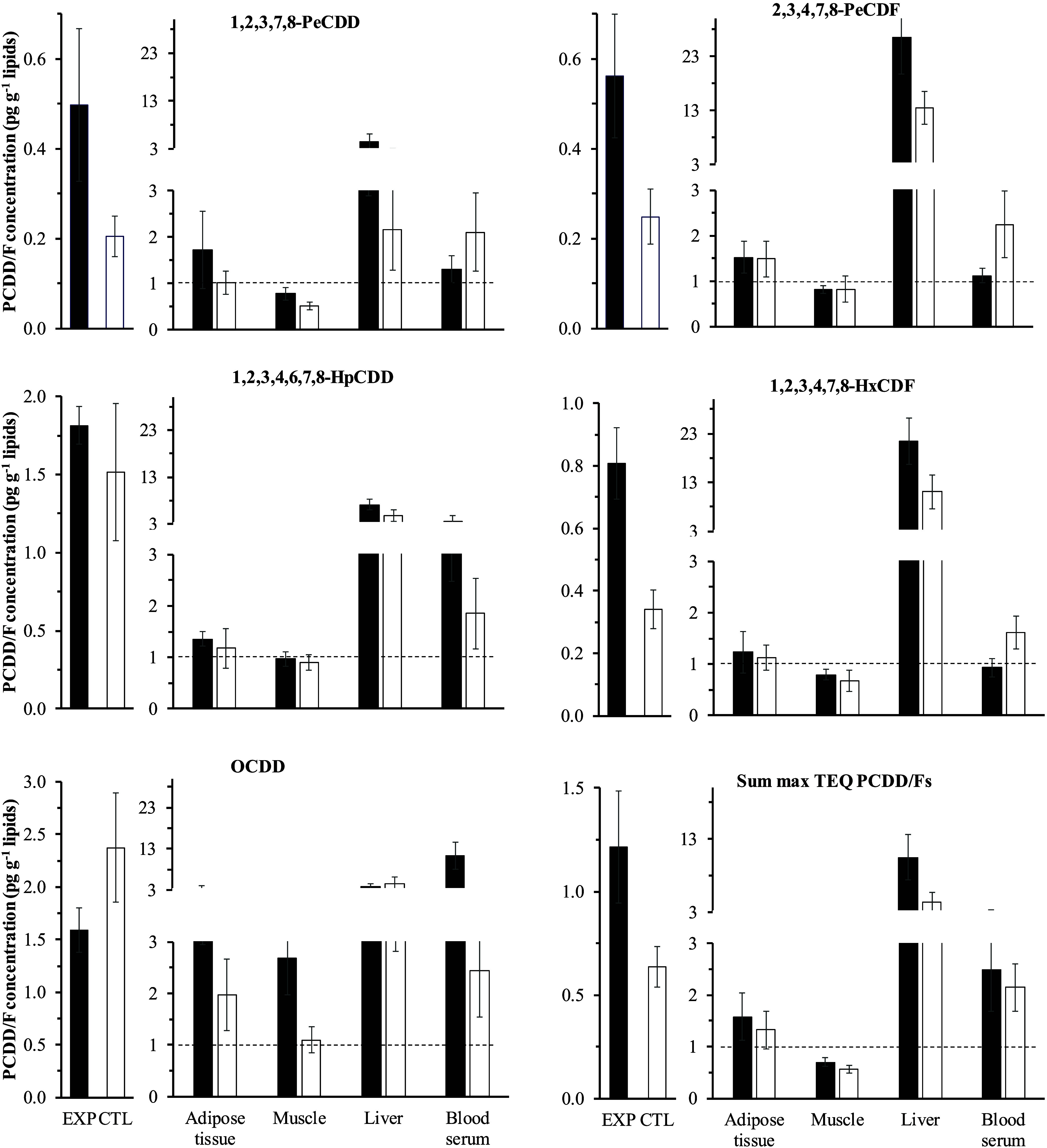
Polychlorinated dibenzo-*p*-dioxin and dibenzofuran (PCDD/F) whole empty body concentrations (leftmost) and, to the right, concentrations in different body tissues normalized to the ones in empty body in depurated (EXP, *n* = 5) and control (CTL, *n* = 4) ewes at slaughter (day 188 or depuration).

**Figure 6:**
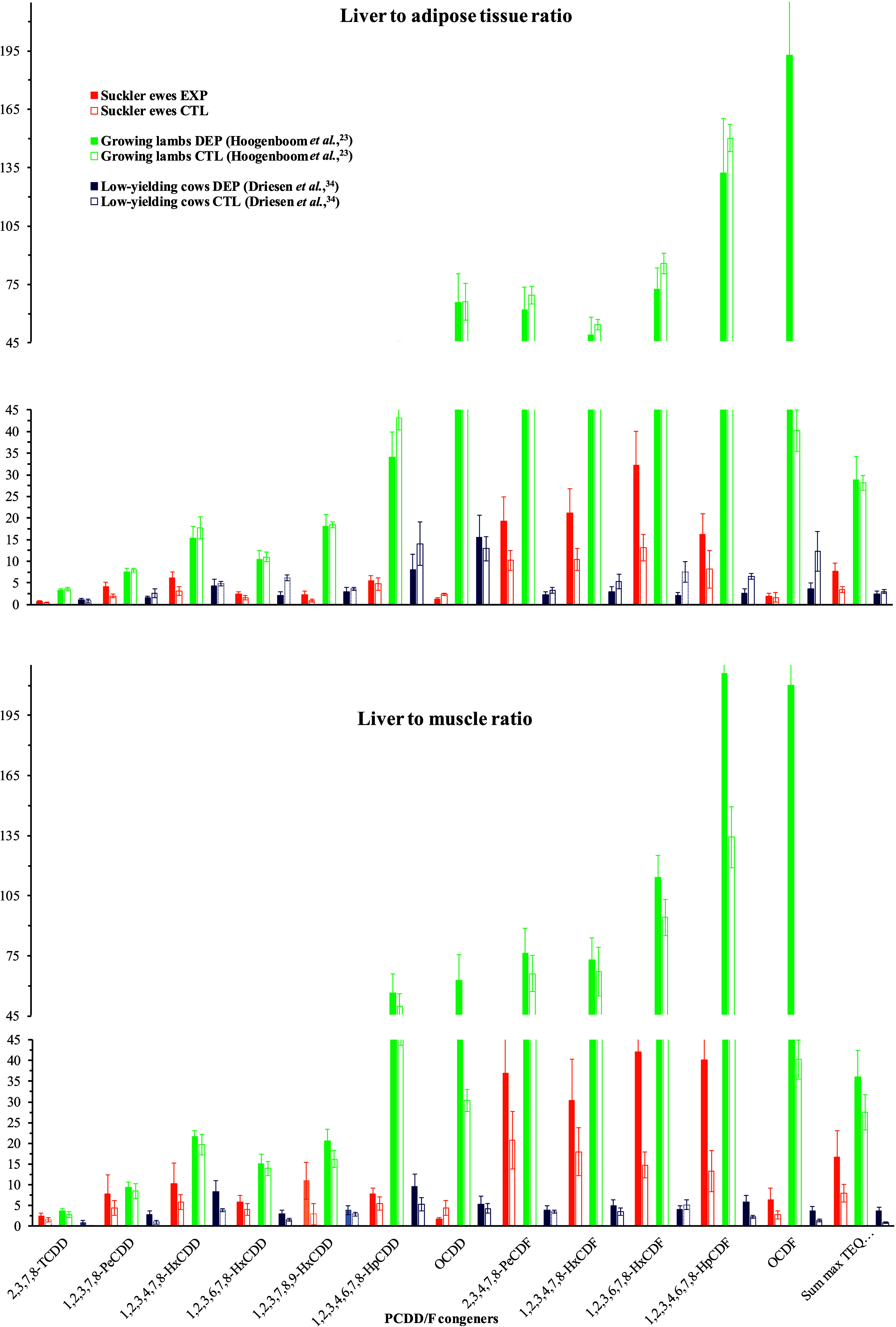
Ratios of liver to adipose tissue or *Longissimus thoracis* muscle lipid-based concentrations in polychlorinated dibenzo-*p*-dioxins and dibenzofurans (PCDD/Fs) in depurated (EXP, *n* = 5) and control (CTL, *n* = 4) ewes (present study at slaughter day 188), compared to lambs (Hoogenboom et al.,^23^ control, CTL or depurated, DEP for 57 days, *n* = 4 each) and lactating cows (Driesen et al.,^34^ control, CTL or depurated, DEP for 124 days, *n* = 4 each). Histograms in light color indicate measurements lower than the limit of detection.

Empty body lipid-based concentrations and body tissue concentrations normalized to the ones in the empty body for five illustrative PCDD/F congeners and in sum TEQ are shown in Figure 5, with numerical results in Supporting Information, Table S10. The ratios of liver to adipose tissue or LT muscle PCDD/F concentrations are shown in Figure 6. Empty body chemical composition and PCDD/F burdens are reported in Supporting Information, Table S11. At slaughter, after 188 days of depuration, the contamination level of the EXP ewes’ empty body was half the ML for meat (1.2 vs 2.5 pg TEQ g^–1^ lipids), but it still tended to be higher than in the CTL ewes (0.6 pg TEQ g^–1^ lipids, *p* = 0.06). The congener pattern in the empty body was similar to that of milk and adipose tissue over the depuration period, that is, a sum TEQ dominated by 1,2,3,7,8-PeCDD (41 and 33% for EXP and CTL ewes). For most congeners, higher concentrations (*p* < 0.05 for 2,3,7,8-TCDD, 1,2,3,6,7,8-HxCDD, 1,2,3,7,8,9-HxCDD, 2,3,7,8-TCDF, 1,2,3,4,6,7,8-HpCDF and OCDF) were recorded in sternal adipose tissue than in the empty body, the reverse being observed for LT muscle (on average for EXP and CTL: 1.3 and 0.6 for the adipose tissue- and LT muscle-to-empty body ratios in sum TEQ, respectively). A remarkable exception was OCDD, with higher (*p* < 0.05) concentrations in both adipose tissue (2.5-fold) and LT muscle (1.8-fold) than in the empty body. Further, blood and, especially, liver showed much higher PCDD/F concentrations (*p* < 0.01) than the empty body: for the sum TEQ, 2.1- and 2.0-fold in serum and up to 9.9- and 4.1-fold in liver of the EXP and CTL ewes, respectively. In blood serum, the highest differences in concentrations against the ones in the empty body were recorded in the EXP ewes for 1,2,3,7,8,9-HxCDD and OCDD (18.1- and 11.4-fold, respectively), followed by 2,3,7,8-TCDD, 1,2,3,6,7,8-HxCDD, 1,2,3,4,7,8,9-HpCDF, and OCDF (5.3-, 5.5-, 5.8-, and 6.6-fold, respectively). In the liver, PCDFs, with the exception of 2,3,7,8-TCDF and OCDF, were higher (*p* < 0.001) in the EXP ewes (29-fold higher than in the empty body, on average, for the six congeners > LOD), as well as in the CTL ewes, to a somewhat lower extent (10-fold higher). Among PCDDs, hexa- and hepta-chlorinated congeners were moderately higher (*p* < 0.05) in the liver than in the empty body (7.4-fold and 2.8-fold higher for the four congeners, in the EXP and CTL ewes, respectively, Figure 5 and Supporting Information, Table S10). Together, this led to 32- and 42-fold higher 1,2,3,6,7,8-HxCDF concentrations in the liver than in adipose tissue and LT muscle, respectively, in the EXP ewes (Figure 6).

At slaughter, the empty body lipid mass and proportions were not different between treatments (*p* ≥ 0.71) and reached, on average, 11.3 kg and 23.5% for the EXP and CTL ewes, respectively. Accordingly, PCDD/F body burdens were approximately twice as high in the EXP than in the CTL ewes (13.8 and 7.2 ng TEQ, respectively, *p* = 0.15, Supporting Information, Table S11). Conversely, the EXP ewe that died at the beginning of the depuration period (day 3) was much leaner, with only 2.1 kg of lipids in the empty body (7.4%), but it had a numerically much higher PCDD/F body burden (74.4 ng TEQ, Supporting Information, Table S3) that was still dominated by 1,2,3,7,8-PeCDD (53% of the sum TEQ vs 41 and 33% in the EXP and CTL ewes on day 188).

## Discussion

Understanding the accumulation and depuration kinetics of environmental contaminants in farm animals is a critical step in ensuring the chemical safety of food. Novel aspects of the current investigation include the investigation of the oral exposure to food toxicokinetics and tissue distribution of the 17 highly toxic and regulated 2,3,7,8-chloro-substituted PCDD/Fs in suckler ewes. For this purpose, we studied a real case of a suckling sheep herd reared extensively in an urban area contaminated with PCDD/Fs due to soil pollution from an old municipal waste incinerator.^7^

### Content, Pattern, and Source of PCDD/Fs in Contaminated Hay

A similar PCDD/F congener pattern was found in all three pools of contaminated hay offered to the EXP ewes over the pre-experimental exposure period, all being dominated in the sum TEQ by 1,2,3,7,8-PeCDD (38 ± 3%) and 2,3,4,7,8-PeCDF (11 ± 2%). This congener pattern was very similar to the one observed in the topsoils of the Lausanne area (contributions to the sum TEQ of 37% for 1,2,3,7,8-PeCDD and 12% for 2,3,4,7,8-PeCDF),^7^ which suggests that hay soiling was most likely the primary source of its PCDD/F contamination, rather than contemporary atmospheric deposition. This finding is supported by the 6-fold higher level of soil impurities in pool 3 compared to pools 1 and 2 of contaminated hay, with hay pool 3 being accordingly 5-fold higher in PCDD/F concentration (12.7 ng TEQ kg^–1^ DM) than hay pools 1 and 2 (2.5 ± 0.4 ng TEQ kg^–1^ DM). Assuming that attached soil was the only source of PCDD/F in contaminated hay and based on soil impurities and PCDD/F concentrations measured in the contaminated hay pools, an expected soil PCDD/F level of approximately 350–610 ng TEQ kg^–1^ DM was estimated; this estimate is remarkably in the 200–640 ng TEQ kg^–1^ DM reported in the Lausanne soil contamination mapping^7^ at the location of the permanent grassland where the contaminated hay was harvested (GPS decimal degree coordinates: 46.52969, 6.63705). When compared to the contaminated hay PCDD/F pattern, milk and adipose tissue patterns showed a slightly higher contribution of 1,2,3,7,8-PeCDD and 2,3,4,7,8-PeCDF to the sum TEQ, in accordance with the higher accumulation rates (characterized by BTFs) of those penta-chlorinated congeners, when compared to hexa-, hepta-, and octa-chlorinated ones.

### Accumulation Rates

Empirical transfer factors are a way to quantify the bioaccumulation potential of contaminants in the feed-to-food chain.^11,12^ The physicochemical properties of individual PCDD/F congeners widely affect their respective BTFs in the present study in ewe, as previously reported for transfer rates or transfer factors in several terrestrial farm animals by Travis and Arms^38^ and Amutova et al.^11^ We observed a clear decreasing trend in BTF from the low-chlorinated and moderately lipophilic (characterized by the partition coefficient between octanol and water, *K*_ow_) tetra- and penta-congeners to the highly chlorinated and highly lipophilic hepta- and octa-congeners. A similar correlation was reported for oral intake-to-milk transfer rates of PCDD/Fs in cows^14−16,39^ and goats.^22^ Further, in growing lambs, adipose tissue BTFs decreased according to the PCDD/F chlorination degree,^23^ within a range and with average values in remarkable agreement with the ones in the ewes of the present study (range 0.01–2.39, mean ± SD: 0.87 ± 0.92 in lambs, vs 0.10–2.65, 0.66 ± 0.71 in ewes; Figure 4). This correlation between transfer factors and their lipophilicity may be explained by the fact that when the log *K*_ow_ exceeds 6.5, the absorption rate decreases sharply in the ruminant digestive tract due to the inability of highly lipophilic molecules to passively diffuse across the unstirred water layer that boards the intestine wall microvilosities.^40,41^ The only exceptions were 1,2,3,7,8-PeCDF, with unexpectedly low BTF when compared to 1,2,3,7,8-PeCDD and 2,3,4,7,8-PeCDF, as well as 2,3,7,8-TCDF and 1,2,3,7,8,9-HxCDF, which were not detected in milk or adipose tissue. Similar low bioaccumulation rates were reported for cows,^14−16,39^ goats,^22^ and lambs^23^ for these three dibenzofuran congeners, which are further suspected to be more efficiently eliminated by metabolic clearance than their equally chlorinated counterparts.

### Depuration Kinetics

Biexponential decay models are commonly used to describe the depuration of lipophilic POPs in milk.^13,15,42^ A biexponential decay model is known to capture depuration from a fast central compartment (blood and organs in fast equilibrium with blood) coupled to a reservoir with slow exchange with blood (mainly adipose tissue). As no measurement was performed between days 0 and 30 (covering the initial fast exponential decay), a monoexponential model was used *ad hoc* in the present study. For interspecies comparison, we also fitted low-yielding cow milk data on days 0, 35, and 63 of depuration^15^ and high-yielding cow data on days 0, 28, and 42 of depuration^14^ to monoexponential models. The depuration kinetics were fitted for adipose tissue data in low-yielding cows on days 0 and 124,^15^ and in growing lambs on days 0 and 57^23^ (Table 2). Contrary to the BTFs, we found no clear trend in the effect of the degree of chlorination on the depuration half-life for the 12 PCDD/F congeners investigated. Nevertheless, on average, slightly longer depuration half-lives were consistently observed for dibenzofurans than for dibenzo-*p*-dioxins in cattle and sheep studies. Milk monoexponential half-lives were on average 1.8-fold longer in low-yielding cows^15^ but 1.3-fold shorter in high-yielding cows^14^ than in the EXP ewes of the present study. Such discrepancies may be partly due to the fact that the EXP ewes in this study and high-yielding cows^14^ excreted more milk lipids per body lipid mass than the low-yielding cows in Driesen et al.,^15,34^ making milk a more effective excretion pathway in ewes and high-yielding cows. Alternatively, this may point to a higher ewe and high-yielding cow hepatic metabolic rate. Indeed, a higher milk production level is concomitant with a higher blood perfusion flow to the liver, as well as a higher hepatic metabolism.^43^

Adipose tissue depuration half-lives were on average 2.1-fold longer than in milk of the EXP ewes for the 12 PCDD/F congeners investigated (*p* < 0.05 for 9 congeners). For low-yielding lactating cows,^15^ a similar situation was found, with half-lives in adipose tissue 3.2-fold longer than in milk. This difference in half-lives between adipose tissue and milk is mechanistically expected, given the slow exchange rate of adipose tissue with blood. At the beginning of the depuration phase, one would expect the concentration in blood (and consequently in milk) to drop sharply, as most of the substances found in nonadipose tissues are rapidly excreted via milk. By contrast, contaminants in adipose tissue must first diffuse slowly into the blood compartment, from which they may then be excreted via milk. Another way of explaining the observed difference in half-lives between adipose tissue and milk is by understanding how the monoexponential curves fitted here approximate actual biexponential behavior (Supporting Information, Section S2). Due to the slow exchange of contaminants between adipose tissue and blood, the dilution of PCDD/Fs caused by an increasing adipose tissue lipid mass over the depuration course is likely an important driver of the decline in adipose tissue PCDD/F concentrations. In agreement, adipose tissue half-lives were longer in low-yielding cows (on average 116 days),^15^ than in ewes (52 days, present study), whereas the former experienced a moderate 1.2-fold increase in estimated body lipid mass along their depuration, compared to 3.4-fold in the EXP ewes. Furthermore, growing lambs showed average adipose tissue half-lives of 64 days for the same PCDD/F congeners,^23^ in close agreement with the EXP ewe values, with a presumably comparable increase in body lipid mass along the depuration in both ewes and lambs (no estimate of body lipid mass was made available in lambs, while lamb BW increased by 1.3-fold vs 1.2-fold in the EXP ewes). A similar dilution effect in adipose tissue was outlined in growing cattle for PCBs,^18^ as well as in calves for PCDD/Fs and PCBs.^15^ The reverse was also observed in exposed^44^ or depurated^45,46^ nonlactating ewes, that is, an increase in adipose tissue concentration of PCBs 138, 153 and 180,^44^ or TCDD and PCBs 126 and 153,^45,46^ due to a decrease in body lipid mass following undernutrition.

### Consequences for Consumer Health Risk Analysis

The respective transfer of PCDD/Fs into milk and edible tissues (i.e., meat encompassing muscles and adipose tissues and offal, such as liver) of sheep has consequences on consumer health risk assessment. Relative to other tissues, a clear enrichment of PCDD/Fs was found in the liver, especially in dibenzofuran congeners. Liver sequestration may result from specific hepatic cytochrome P450-binding.^24^ Such enrichment, characterized by the liver to adipose tissue ratio in PCDD/F concentrations was already reported in lactating cows, but at a rather lower rate,^34^ whereas it was found to be higher in growing lambs (Figure 6).^23^ A lower metabolic activity of cytochrome P450 enzymes in sheep was identified as a possible explanation for higher PCDD/F levels in sheep liver.^24^ When compared to LT muscle, PCDD/F concentrations were higher in the sternal adipose tissue (2.3-fold for the sum TEQ). A similar observation was made for LT muscle and subcutaneous adipose tissue of depurated lactating cows and growing calves.^34^ Such a discrepancy among tissue concentrations warrants caution for food safety monitoring. Indeed, the PCDD/F content measured in a specific tissue is not necessarily representative of all of the others.

In conclusion, the present toxicokinetic study highlighted a relevant PCDD/F transfer from oral intake to milk and adipose tissue in ewes, especially for penta- and hexa-chlorinated congeners. It also confirms that it is feasible to depurate ewe adipose tissue under the EU and Swiss ML over a time period of 130 days, starting with an initial PCDD/F level approximately 10-fold the ML. Milk depuration was even faster, and according to the derived depuration half-life, milk would be compliant with the EU and Swiss ML (2.0 pg g^–1^ lipids) after approximately 80 days of depuration starting from approximately 14-fold the ML. Nevertheless, such a theoretical milk concentration compliant with the ML was never reached in the present study, since ewes were no longer lactating after 60 days of depuration (weaning time). During lactation, depuration probably occurred mainly through excretion of PCDD/Fs via milk lipids. After weaning, ewe adipose tissue concentrations continued to decrease steadily. Over such a period, excretion was limited to fecal output, while the dilution effect from increasing body lipid mass likely played a major role in the decline in adipose tissue concentration. In practice, the feasibility to depurate a contaminated herd will additionally depend on the availability of noncontaminated forages for feeding depurated animals, as well as on the amount of time available to recover levels lower than the milk, meat or liver ML, which should in turn be concordant with the production cycle time frame in order to remain economically viable for the farmer. A specific accumulation of PCDD/Fs was observed in the ewe liver, especially for dibenzofurans. The toxicokinetic data presented here will be useful in setting up physiologically based toxicokinetic models quantifying the fate of PCDD/Fs in lactating ewes. Such models have proven to be very useful in risk assessment and risk management activities to protect consumers from negative health effects from chronic exposure to environmental contaminants from foods of animal origin.^42,47−49^

## Supporting Information Available

Details on test for differences in half-lives between milk and adipose tissue (Section S1); mechanistic reason for difference in half-life between adipose tissue and milk (Section S2); physiological traits (Table S1) and tissue PCDD/F concentrations and body burdens (Table S2) of the EXP ewe died on depuration day 3; chemical composition of hays and concentrate feeds (Table S3); analytical LOD for PCDD/F concentrations (Table S4); kinetics in ewe physiological traits (Table S5) and blood metabolites (Table S6); PCDD/F depuration kinetics in ewe milk (Table S7), blood serum (Table S8) and sternal adipose tissue (Table S9); ewe PCDD/F tissue distribution at slaughter (depuration day 188, Table S10); and slaughter empty body PCDD/F burdens (Table S11) (PDF)

## Abbreviations Used

APGC-MS: atmospheric pressure gas chromatography coupled with mass-spectrometry
BTF: biotransfer factor
BW: body weight
CH-TAX: Swiss cattle and sheep carcass grading classification scheme
CTL: control treatment and ewes
DM: dry matter
EU: European Union
EXP: exposed and further depurated experimental treatment and ewes
INRA: French “Institut National de la Recherche Agronomique”
*K*_ow_: partition coefficient between octanol and water
LT: *Longissimus thoracis* muscle
LOD: limit of detection
ML: regulatory maximum level
PCB: polychlorinated biphenyl
PCDD/F: polychlorinated dibenzo-*p*-dioxin and furan
POP: persistent organic pollutant
*R*^2^: coefficient of determination
rCV: residual coefficient of variation
RMSE: root-mean-square error
SD: standard deviation
SEM: standard error of the mean
TEQ: toxic equivalent
