# Supplementary material for: Accumulation Rate, Depuration Kinetics, and Tissue Distribution of Polychlorinated Dibenzo-p-Dioxins and Dibenzofurans (PCDD/Fs) in Suckler Ewes (Ovis aries)
Source: J Agric Food Chem. 2024 Jun 17;72(26):14941–55. doi: 10.1021/acs.jafc.4c02626 (PMC11228998; doi:10.1021/acs.jafc.4c02626)
Supplement: Supplementary file 1 — jf4c02626_si_001.pdf [file jf4c02626_si_001.pdf]

**SUPPORTING INFORMATION**

# Accumulation rate, depuration kinetics, and tissue distribution of polychlorinated dibenzo-*p*-dioxins and dibenzofurans (PCDD/Fs) in suckler ewes (*Ovis aries*)

Sylvain Lerch[1,*], Raphaël Siegenthaler[2], Jorge Numata[3], Jan-Louis Moenning[3], Frigga Dohme-Meier[1] and Markus Zennegg[4]

[1]Ruminant Nutrition and Emissions, Agroscope, 1725 Posieux, Switzerland

[2]Research Contracts Animals, Agroscope, 1725 Posieux, Switzerland

[3]Department Safety in the Food Chain, German Federal Institute for Risk Assessment (BfR), Max-Dohrn-Str. 8-10, 10589 Berlin, Germany

[4]Laboratory for Advanced Analytical Technologies, Empa, Überlandstrasse 129, 8600 Dübendorf, Switzerland

* Corresponding author:

Sylvain Lerch, Agroscope, Route de la Tioleyre 4, 1725 Posieux, Switzerland; sylvain.lerch@agroscope.admin.ch; Tel. 0041 58 461 41 29; ORCID: 0000-0003-0957-8012

**Sections S1-S2, Tables S1-S11 and Figure S1**

**Pages S2-S14**

## Section S1

### Test for differences in half-lives between milk and adipose tissue

To statistically assess the difference in half-life between milk and adipose tissue for each congener, the one sample *t*-test was used. To assess the difference in half-lives, differences in the exponential parameter *k* (related by eq [4]) are actually tested, since it is *k* that is assumed to be normally distributed (Lam et al., 1985).

The *t*-test requires an estimate of the variance, for which the delete one Jackknife method was employed. In addition to using all 5 ewes at once to derive the slope parameter $k$, this was also done with all 5 possible subsets of 4 ewes deriving parameters $k_{adipose,i}$ and $k_{milk,i}$ describing the slope induced by only 4 ewes omitting ewe $i$. Next, the difference of the main sample and the Jackknife samples were determined i.e., $\Delta k_i = k_{adipose\,,i} - k_{milk,i}$ and $\Delta k = k_{adipose} - k_{milk}$. The variance of $\Delta k$ can now be determined from $\Delta k_i$ as follows:

First determine

$$\overline{\Delta k} = \frac{1}{5}\sum_{i=1}^{5} \Delta k_i. \quad \text{(S1)}$$

Then the variance is given by

$$\sigma_\Delta^2 = 4\sum_{i=1}^{5}\left(\overline{\Delta k} - \Delta k_i\right)^2. \quad \text{(S2)}$$

Finally, the test static for the one sample t-test is given by

$$t = \frac{\sqrt{n}\Delta k}{\sigma_\Delta}. \quad \text{(S3)}$$

### Confidence intervals for the estimated half-lives

The confidence interval for each congener's half-life (separately for adipose tissue and milk) is calculated from the confidence interval of the parameter k estimates using the Jackknife method by determining $k_{adipose\,,i}$ and $k_{milk,i}$ , i.e., first determine

$$\overline{k_j} = \frac{1}{5}\sum_{i=1}^{5} k_{j,i}, \quad \text{(S4)}$$

where *j* ∊ {milk,adipose}. Then determine the variance of $k_j$ as

$$\sigma_j^2 = 4\sum_{i=1}^{5}\left(\overline{k_j} - k_{j,i}\right)^2. \quad \text{(S5)}$$

The confidence interval (95%) is given by $k_j \pm 1{,}96\frac{\sigma_j}{\sqrt{5}}$. The confidence intervals for each half-life is then given by $[\frac{\ln(2)}{k_j+1{,}96\frac{\sigma_j}{\sqrt{5}}}, \frac{\ln(2)}{k_j-1{,}96\frac{\sigma_j}{\sqrt{5}}}]$.

Lam, F. C.; Hung, C. T.; Perrier, D. G., Estimation of variance for harmonic mean half-lives. *Journal of Pharmaceutical Sciences* **1985**, *74* (2), 229-231.

## Section S2

### Mechanistic reason for the difference in half-life between adipose tissue and milk

Figure S1 shows a plot with typical depuration phase with concentration-time behaviour in adipose tissue (blue) and blood (red) for lipophilic substances with biphasic (bi-exponential) behaviour. This plot comes from a generic two-compartment model with a slow exchange between the central (blood and fast exchange tissues and secretions) and peripheral (mainly adipose) compartments. The behaviour for milk is very similar to blood because of the fast exchange between milk and blood (so the red plot in Supporting Figure S1 is tantamount to milk). In this work on suckler ewes, we had no experimental information about the beginning of the depuration phase, which forced the use of mono-exponential curves to fit each of the naturally biexponential behaviours. From this plot, it becomes clear that a mono-exponential (a straight line in this semilog-y plot) can approximate the behaviour in adipose tissue relatively well; a mono-exponential fitted half-life for adipose tissue will thus be close to the actual behaviour of adipose tissue. However, for blood (and thus milk), it will overestimate the half-life at the beginning (thus underestimating the decay speed) and vice versa towards the end. A mono-exponential half-life will thus, in general, not closely reflect the behaviour of blood, liver or milk; the fitted mono-exponential half-life for milk is expected in general to be lower (have a steeper line in the semilog-y plot) than the fitted mono-exponential adipose half-life, which is what we observed in the experiment.

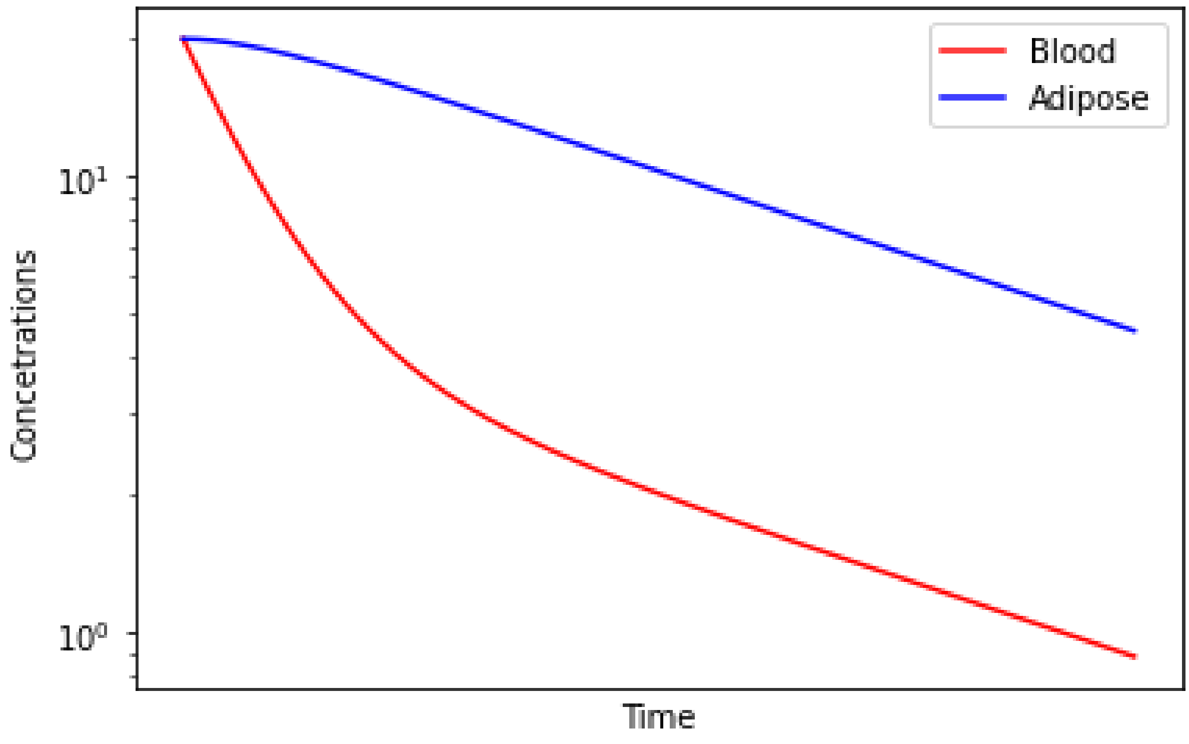


**Figure S1**. Caricature plot showing typical depuration phases in adipose tissue (blue) and blood (red) for lipophilic substances with biphasic (bi-exponential) behaviour. This plot comes from a generic two-compartment model with constant compartment sizes.

**Table S1.** Body traits and chemical composition of the ewe of the experimental (EXP) treatment died on day 3 of experiment (end of exposure period).

| | |
|---|---|
| Body weight (kg) | 41.2 |
| Empty body weight (kg) | 28.1 |
| Carcass weight (kg) | 19.8 |
| Perirenal adipose tissue weight (kg) | 0.056 |
| Body fat score (1-5)[a] | 1 |
| Body conformation score (1-5)[a] | 1 |
| Sternal adipose cell diameter (µm) | 35.7 |
| Subcutaneous adipose tissue thickness (cm)[b] | 0.12 |
| Empty body chemical component masses (kg) | |
| Water | 19.9 |
| Lipids | 2.1 |
| Proteins | 5.1 |
| Minerals | 1.5 |
| Empty body chemical component proportions (%) | |
| Water | 70.7 |
| Lipids | 7.4 |
| Proteins | 18.2 |
| Minerals | 5.3 |

[a]According to the Swiss CH-TAX carcass classification system (Order 916.341.22 of Swiss federal laws, 1999, last update 2003). For conformation score the following numerical conversion was performed: C = 5, H = 4, T+ = 3.5, T = 3, T- = 2.5, A = 2 and X = 1.
[b]Measured by ultrasound (Logiq e R7 US equipped with a 9L-RS Linear-Transducer, 3.0-9.0 MHz; GE Healthcare, Glattbrugg, Switzerland) between the 4$^{th}$ and 5$^{th}$ lumbar vertebras.

**Table S2.** Tissue distribution and empty body burden of polychlorinated dibenzo-*p*-dioxins and dibenzofurans (PCDD/Fs) of the ewe of the experimental (EXP) treatment died on day 3 of experiment (end of exposure period).

| | Tissue concentration (pg g$^{-1}$ lipids) | | | | | | |
|---|---|---|---|---|---|---|---|
| PCDD/F congener | Milk | Empty body | Sternal adipose tissue | *Longissimus thoracis* muscle | Liver | Blood serum | Empty body burden (ng) |
| 2,3,7,8-TCDD | 3.23 | 3.25 | 3.41 | 2.18 | 5.52 | 4.44 | 6.72 |
| 1,2,3,7,8-PeCDD | 26.16 | 19.02 | 24.77 | 14.55 | 55.48 | 16.65 | 39.30 |
| 1,2,3,4,7,8-HxCDD | 25.73 | 17.34 | 26.37 | 12.22 | 90.94 | 5.73 | 35.85 |
| 1,2,3,6,7,8-HxCDD | 32.71 | 17.79 | 34.26 | 16.89 | 80.44 | 5.62 | 36.77 |
| 1,2,3,7,8,9-HxCDD | 14.59 | 7.94 | 13.26 | 7.41 | 42.51 | 6.32 | 16.40 |
| 1,2,3,4,6,7,8-HpCDD | 31.82 | 39.82 | 75.27 | 29.85 | 402.19 | 48.58 | 82.30 |
| OCDD | 7.58 | 15.66 | 240.66 | 19.73 | 214.70 | 64.17 | 32.37 |
| 2,3,7,8-TCDF | 0.09 | 1.83 | 0.63 | 0.28 | 0.30 | 5.35 | 3.78 |
| 1,2,3,7,8-PeCDF | 0.50 | 0.87 | 1.61 | 0.27 | 0.26 | 5.47 | 1.79 |
| 2,3,4,7,8-PeCDF | 22.99 | 16.07 | 19.31 | 11.86 | 313.62 | 19.48 | 33.22 |
| 1,2,3,4,7,8-HxCDF | 29.93 | 16.00 | 24.53 | 12.84 | 301.98 | 6.44 | 33.07 |
| 1,2,3,6,7,8-HxCDF | 20.49 | 11.86 | 15.37 | 9.44 | 323.51 | 15.43 | 24.52 |
| 1,2,3,7,8,9-HxCDF | | | | < LOD | | | |
| 2,3,4,6,7,8-HxCDF | 14.43 | 9.25 | 11.43 | 7.03 | 287.59 | 6.38 | 19.12 |
| 1,2,3,4,6,7,8-HpCDF | 17.91 | 24.14 | 27.88 | 13.48 | 706.84 | 30.35 | 49.90 |
| 1,2,3,4,7,8,9-HpCDF | 1.36 | 1.67 | 2.24 | 1.38 | 54.23 | 9.80 | 3.45 |
| OCDF | 0.50 | 2.94 | 17.87 | 1.35 | 49.24 | 12.28 | 6.07 |
| Sum Raw min | 249.9 | 196.4 | 538.3 | 160.8 | 2931.1 | 201.1 | 406.0 |
| Sum Raw max | 250.2 | 205.8 | 539.8 | 161.2 | 2931.1 | 274.7 | 425.3 |
| Sum TEQ min[a] | 50.63 | 35.20 | 47.67 | 27.35 | 279.70 | 24.85 | 72.76 |
| Sum TEQ max[a] | 50.60 | 36.01 | 47.83 | 27.39 | 279.70 | 34.36 | 74.43 |

In gray the measurement lower than the limit of detection (LOD) are reported as equal to LOD.
[a]Results normalized for TEQ are determined according to the WHO 2005 TEF values.[35]

**Table S3.** Feed nutritional composition.

| Nutrient (% dry matter) | Contaminated hay pool 1 (11/01/21 – 02/01/22) | Contaminated hay pool 2 (02/15/22 – 03/03/22) | Contaminated hay pool 3 (03/08/22 – 03/29/22) | Non-contaminated hay n°1 (11/01/21-02/14/22)[a] | Non-contaminated hay n°2 (02/15/22-11/09/22)[a] | Complete concentrate feedstuff[a,b] | Pelleted whole maize plant[a] |
|---|---|---|---|---|---|---|---|
| Organic matter | 92.5 | 93.6 | 90.1 | 93.2 | 92.9 | 93.3 | 96.0 |
| Crude proteins | 8.5 | 6.7 | 7.6 | 8.7 | 7.4 | 17.3 | 5.7 |
| Neutral-detergent fiber | 60.1 | 65.2 | 59.7 | 61.7 | 63.5 | 22.2 | 43.0 |
| Acid-detergent fiber | 36.9 | 38.0 | 35.0 | 37.6 | 37.2 | 10.6 | 23.6 |
| Starch | ND | ND | ND | ND | ND | 37.7 | 31.9 |
| Ether-extracted fat[c] | 1.6 | 1.6 | 1.6 | 1.7 | 1.7 | 6.3 | 2.7 |
| Acid-insoluble ashes | 2.9 | 2.5 | 5.5 | 2.0 | 2.3 | 0.2 | 0.9 |
| Soil impurities | 0.79 | 0.37 | 3.58 | 0.48 | 0.91 | 1.48 | 0.09 |

ND: not determined.
[a]Mean of three successive pools.
[b]Sheep and goat organic feedstuff 16% crude protein, n°4785.2A, Anitech Moulin Chevalier SA, Cuarnens, Switzerland.
[c]With previous acid-hydrolysis.

**Table S4.** Limit of detection (LOD) from the means of the procedural blanks per matrix.[a]

| Matrix | Blood serum throughout experiment | Milk throughout experiment | Subcutaneous adipose tissue from biopsy | Muscle from slaughter | Liver from slaughter | Empty body homogenate from slaughter |
|---|---|---|---|---|---|---|
| Extraction | Liquid-liquid | Liquid-liquid | Mortar | Soxhlet | Soxhlet | Soxhlet |
| Sample intake (kg) | 0.0005 | 0.003 | 0.001 | 0.002 | 0.003 | 0.004 |
| 2,3,7,8-TCDD | 0.30 | 0.10 | 0.25 | 0.10 | 0.050 | 0.030 |
| 1,2,3,7,8-PeCDD | 0.40 | 0.10 | 0.30 | 0.10 | 0.050 | 0.040 |
| 1,2,3,4,7,8-HxCDD | 0.40 | 0.30 | 0.35 | 0.10 | 0.050 | 0.040 |
| 1,2,3,6,7,8-HxCDD | 0.40 | 0.30 | 0.35 | 0.10 | 0.050 | 0.040 |
| 1,2,3,7,8,9-HxCDD | 0.40 | 0.40 | 0.35 | 0.10 | 0.050 | 0.040 |
| 1,2,3,4,6,7,8-HpCDD | 0.90 | 0.90 | 0.40 | 0.20 | 0.15 | 0.10 |
| OCDD | 0.30 | 0.40 | 3.0 | 0.30 | 0.20 | 0.15 |
| 2,3,7,8-TCDF | 0.50 | 0.10 | 0.40 | 0.15 | 0.070 | 0.040 |
| 1,2,3,7,8-PeCDF | 0.50 | 0.10 | 0.40 | 0.20 | 0.070 | 0.040 |
| 2,3,4,7,8-PeCDF | 0.50 | 0.10 | 0.40 | 0.20 | 0.070 | 0.040 |
| 1,2,3,4,7,8-HxCDF | 0.70 | 0.30 | 0.40 | 0.20 | 0.080 | 0.050 |
| 1,2,3,6,7,8-HxCDF | 0.70 | 0.30 | 0.40 | 0.20 | 0.080 | 0.050 |
| 1,2,3,7,8,9-HxCDF | 0.70 | 0.30 | 0.40 | 0.20 | 0.080 | 0.050 |
| 2,3,4,6,7,8-HxCDF | 0.70 | 0.30 | 0.40 | 0.20 | 0.080 | 0.050 |
| 1,2,3,4,6,7,8-HpCDF | 1.0 | 0.50 | 0.60 | 0.25 | 0.10 | 0.090 |
| 1,2,3,4,7,8,9-HpCDF | 1.0 | 0.50 | 0.60 | 0.25 | 0.10 | 0.090 |
| OCDF | 2.5 | 0.90 | 2.5 | 0.50 | 0.30 | 0.20 |

[a]Mean of several blanks measured parallel to the matrices are displayed. Sample intakes are important to take into account, as LOD is sensitive to different sample volumes and lipid loads.

**Table S5.** Ewe body weight, and indicators of body fatness.[a]

| Item | Treatment | Depuration days |  |  |  |  |  |  | SEM | *p*-value |  |  |
|---|---|---|---|---|---|---|---|---|---|---|---|---|
|  |  | 0 |  | 32 | 60 |  | 130 | 188 |  | Treatment | Day | Trt × Day |
| Body weight (kg) | EXP | 49.9 c | * | 52.6 b | 54.1 b | * | 55.5 b | 58.8 a | 3.27 | 0.07 | < 0.01 | 0.11 |
|  | CTL | 62.2 b |  |  | 64.9 a |  |  | 64.0 a,b |  |  |  |  |
| Body fatness score (1-5)[b] | EXP | 1.2 b |  | 1.2 b | 1.0 b |  | 2.6 a | 2.8 a | 0.27 | 0.28 | < 0.001 | 0.08 |
|  | CTL | 1.0 b |  |  | 1.5 b |  |  | 3.6 a |  |  |  |  |
| Body conformation score (1-5)[b] | EXP | 1.2 b |  | 1.2 b | 1.0 b | * | 2.4 a | 2.2 a | 0.19 | 0.03 | < 0.001 | 0.10 |
|  | CTL | 1.5 b |  |  | 2.0 b |  |  | 2.6 a |  |  |  |  |
| Subcutaneous adipose tissue thickness (cm)[c] | EXP | 0.30 c |  | 0.24 c | 0.27 c |  | 0.55 b | 0.74 a | 0.062 | 0.99 | < 0.001 | 0.46 |
|  | CTL | 0.22 b |  |  | 0.31 b |  |  | 0.78 a |  |  |  |  |
| Sternal adipose cell diameter (µm) | EXP | 56.1 b |  | 56.0 b | 56.0 b |  | 64.0 b | 70.1 a | 5.22 | 0.94 | 0.01 | 0.06 |
|  | CTL | 61.4 |  |  | 58.0 |  |  | 61.2 |  |  |  |  |
| Empty body lipid mass (kg)[d] | EXP | 3.4 b |  | 3.9 b | 3.5 b |  | 9.1 a | 11.5 a | 1.43 | 0.27 | < 0.001 | 0.39 |
|  | CTL | 4.8 b |  |  | 7.0 b |  |  | 12.8 a |  |  |  |  |

a-c within row, least-square means with different letters differ at $p \leq 0.05$.
* At the specific depuration day, EXP and CTL least-square means differ at $p \leq 0.05$.
[a]The five depurated (EXP) ewes were formerly (until day 0) fed with a hay contaminated with PCDD/Fs, and further received a non-contaminated hay until 188 days of depuration. The four control (CTL) ewes were continuously fed with non-contaminated hay. At day 0, ewes were lactating on average at 29 days in milk, weaning / dry-off took place at depuration day 63, and thereafter ewes were non-lactating and non-gestating until the end of the depuration (day 188).
[b]According to the Swiss CH-TAX carcass classification system (Order 916.341.22 of Swiss federal laws, 1999, last update 2003). For conformation score the following numerical conversion was performed: C = 5, H = 4, T+ = 3.5, T = 3, T- = 2.5, A = 2 and X = 1.
[c]Measured by ultrasound (Logiq e R7 US equipped with a 9L-RS Linear-Transducer, 3.0-9.0 MHz; GE Healthcare, Glattbrugg, Switzerland) between the 4$^{th}$ and 5$^{th}$ lumbar vertebras.
[d]According to the estimation eq [1] set at slaughter (day 188): Empty body lipids (kg) = - 9.06 + 0.17 × BW (kg) + 3.37 × Body fatness score (1-5).

**Table S6.** Ewe blood serum lipid classes and beta-hydroxybutyrate contents.[a]

| Blood serum metabolite | | Depuration days | | | | | | *p*-value | | |
|---|---|---|---|---|---|---|---|---|---|---|
| (mg $dL^{-1}$, unless stated) | Treatment | 0 | 32 | 60 | 130 | 188 | SEM | Treatment | Day | Trt × Day |
| Non-esterified fatty acids | EXP | 24.8 a | 6.0 b | 8.0 b | 3.0 c | 4.3 b,c | 2.28 | 0.60 | < 0.001 | 0.26 |
| | CTL | 15.1 a | | 9.3 a | | 2.6 b | | | | |
| Triglycerides | EXP | 14.9 | 13.2 | 14.6 | 10.8 | 13.9 | 2.36 | 0.15 | 0.58 | 0.71 |
| | CTL | 10.8 | | 9.2 | | 12.2 | | | | |
| Free cholesterol | EXP | 17.8 a | 15.3 a,b | 13.7 b,c | 10.8 c | 12.1 b,c | 1.74 | 0.94 | < 0.01 | 0.91 |
| | CTL | 17.8 a | | 13.3 b | | 12.9 b | | | | |
| Cholesteryl-esters | EXP | 124 a,b | 142 a | 123 a,b | 99 c | 103 b,c | 9.4 | 0.19 | < 0.001 | 0.02 |
| | CTL | 167 a * | | 114 b | | 109 b | | | | |
| Phospholipids | EXP | 122 a | 131 a | 114 a,b | 100 b | 99 b | 6.8 | 0.21 | < 0.001 | 0.28 |
| | CTL | 143 a | | 113 b | | 106 b | | | | |
| Free glycerol | EXP | 0.92 a | 0.39 b | 0.48 b | 0.37 b | 0.29 b | 0.082 | 0.13 | < 0.001 | 0.21 |
| | CTL | 0.64 a | | 0.38 b | | 0.29 b | | | | |
| Beta-hydroxybutyrate (µM) | EXP | 386 a | 374 a | 298 a,b | 222 b | 250 b | 41.7 | 0.67 | < 0.01 | 0.50 |
| | CTL | 350 | | 243 | | 289 | | | | |

a-c within row, least-square means with different letters differ at $p \leq 0.05$.

* At the specific depuration day, EXP and CTL least-square means differ at $p \leq 0.05$.

[a]The five depurated (EXP) ewes were formerly (until day 0) fed with a hay contaminated with PCDD/Fs, and further received a non-contaminated hay until 188 days of depuration. The four control (CTL) ewes were continuously fed with non-contaminated hay. At day 0, ewes were lactating on average at 29 days in milk, weaning / dry-off took place at depuration day 63, and thereafter ewes were non-lactating and non-gestating until the end of the depuration (day 188).

**Table S7.** Depuration kinetic of ewe milk concentrations in polychlorinated dibenzo-*p*-dioxins and dibenzofurans (PCDD/Fs).[a]

| PCDD/F congener (pg $g^{-1}$ lipids)[b] | Treatment | Depuration days | | | | | SEM | *p*-value | | |
|---|---|---|---|---|---|---|---|---|---|---|
| | | 0 | | 32 | 60 | | | Treatment | Day | Trt × Day |
| 2,3,7,8-TCDD | EXP | 1.97 a | * | 0.61 b | 0.29 c | * | 0.150 | <0.001 | <0.001 | <0.001 |
| | CTL | 0.08 | | | 0.06 | | | | | |
| 1,2,3,7,8-PeCDD | EXP | 14.50 a | * | 4.46 b | 2.44 c | * | 1.052 | <0.001 | <0.001 | <0.01 |
| | CTL | 0.39 | | | 0.20 | | | | | |
| 1,2,3,4,7,8-HxCDD | EXP | 11.33 a | * | 4.22 b | 2.70 c | * | 0.968 | <0.001 | <0.01 | 0.01 |
| | CTL | 0.34 | | | 0.27 | | | | | |
| 1,2,3,6,7,8-HxCDD | EXP | 13.14 a | * | 5.42 b | 3.24 c | * | 1.097 | <0.001 | <0.001 | 0.04 |
| | CTL | 0.66 | | | 0.40 | | | | | |
| 1,2,3,7,8,9-HxCDD | EXP | 5.58 a | * | 1.10 b | 0.57 b | * | 0.472 | <0.001 | <0.001 | 0.04 |
| | CTL | 0.21 a | | | 0.07 b | | | | | |
| 1,2,3,4,6,7,8-HpCDD | EXP | 11.64 a | * | 3.54 b | 2.16 c | * | 1.025 | <0.01 | <0.001 | 0.07 |
| | CTL | 2.17 a | | | 0.95 b | | | | | |
| OCDD | EXP | 6.74 a | | 2.82 b | 1.36 b | | 1.985 | 0.55 | <0.01 | 0.69 |
| | CTL | 6.14 a | | | 1.43 b | | | | | |
| 2,3,7,8-TCDF | EXP | | | | | < LOQ | | | | |
| | CTL | | | | | | | | | |
| 1,2,3,7,8-PeCDF | EXP | 0.25 a | | 0.08 b | 0.21 a | * | 0.040 | <0.001 | <0.001 | <0.01 |
| | CTL | 0.13 a | | | 0.03 b | | | | | |
| 2,3,4,7,8-PeCDF | EXP | 14.60 a | * | 5.02 b | 2.64 c | * | 0.907 | <0.001 | <0.001 | <0.01 |
| | CTL | 0.45 a | | | 0.22 b | | | | | |
| 1,2,3,4,7,8-HxCDF | EXP | 14.08 a | * | 5.38 b | 3.58 b | * | 1.136 | <0.001 | <0.01 | 0.06 |
| | CTL | 0.54 | | | 0.35 | | | | | |
| 1,2,3,6,7,8-HxCDF | EXP | 9.51 a | * | 3.24 b | 1.96 b | * | 0.600 | <0.001 | <0.01 | 0.06 |
| | CTL | 0.28 a* | | | 0.13 b | | | | | |
| 1,2,3,7,8,9-HxCDF | EXP | | | | | < LOQ | | | | |
| | CTL | | | | | | | | | |
| 2,3,4,6,7,8-HxCDF | EXP | 6.57 a | * | 2.22 b | 1.30 c | * | 0.487 | <0.001 | <0.001 | 0.05 |
| | CTL | 0.27 a | | | 0.11 b | | | | | |
| 1,2,3,4,6,7,8-HpCDF | EXP | 6.75 a | * | 3.18 b | 2.40 b | * | 0.726 | <0.001 | <0.01 | 0.89 |
| | CTL | 1.15 a | | | 0.36 b | | | | | |
| 1,2,3,4,7,8,9-HpCDF | EXP | 0.51 | | 0.29 | 0.30 | * | 0.067 | <0.01 | 0.02 | 0.18 |
| | CTL | 0.28 a | | | 0.10 b | | | | | |
| OCDF | EXP | 0.66 | | 0.52 | 0.39 | | 0.166 | 0.62 | 0.03 | 0.13 |
| | CTL | 0.87 a | | | 0.28 b | | | | | |
| Sum Raw min | EXP | 117.9 a | * | 41.4 b | 24.4 c | * | 8.70 | <0.001 | <0.001 | 0.10 |
| | CTL | 12.5 a | | | 4.8 b | | | | | |
| Sum Raw max | EXP | 118.2 a | * | 42.5 b | 26.1 c | * | 8.62 | <0.001 | <0.001 | 0.21 |
| | CTL | 14.3 a | | | 5.1 b | | | | | |
| Sum TEQ min[c] | EXP | 27.22 a | * | 8.8 b | 4.76 c | * | 1.917 | <0.001 | <0.01 | <0.01 |
| | CTL | 0.71 | | | 0.47 | | | | | |
| Sum TEQ max[c] | EXP | 27.25 a | * | 8.85 b | 4.97 c | * | 1.916 | <0.001 | <0.001 | <0.01 |
| | CTL | 0.91 a | | | 0.49 b | | | | | |

a-c within row, least-square means with different letters differ at $p \leq 0.05$.
* At the specific depuration day, EXP and CTL least-square means differ at $p \leq 0.05$.
In gray the measurement lower than the limit of detection (LOD) are reported as equal to LOD.
[a]The five depurated (EXP) ewes were formerly (until day 0) fed with a hay contaminated with PCDD/Fs, and further received a non-contaminated hay until 188 days of depuration. The four control (CTL) ewes were continuously fed with non-contaminated hay. At day 0, ewes were lactating on average at 29 days in milk, weaning / dry-off took place at depuration day 63, and thereafter ewes were non-lactating and non-gestating until the end of the depuration (day 188).
[b]Upper-bound values (fix to LOD when recorded at level < LOD), unless for Sum Raw min and Sum TEQ min where LOD values are set to 0.
[c]Results normalized for TEQ are determined according to the WHO 2005 TEF values.[35]

**Table S8.** Depuration kinetic of ewe blood serum concentrations in polychlorinated dibenzo-*p*-dioxins and dibenzofurans (PCDD/Fs).[a]

| PCDD/F congener (pg g$^{-1}$ lipids)[b] | Treatment | Depuration days | | | | |
|---|---|---|---|---|---|---|
| | | 0 | 32 | 60 | 130 | 188 |
| 2,3,7,8-TCDD | EXP | 0.33 | 1.27 | 0.44 | 0.57 | 0.49 |
| | CTL | 0.54 | | 1.57 | | 0.24 |
| 1,2,3,7,8-PeCDD | EXP | 7.16 | 6.23 | 1.30 | 1.13 | 0.53 |
| | CTL | 0.97 | | 2.74 | | 0.39 |
| 1,2,3,4,7,8-HxCDD | EXP | 8.48 | 6.64 | 2.04 | 1.22 | 0.06 |
| | CTL | 0.91 | | 3.25 | | 0.04 |
| 1,2,3,6,7,8-HxCDD | EXP | 9.29 | 10.50 | 2.53 | 1.15 | 0.35 |
| | CTL | 1.01 | | 3.62 | | 0.04 |
| 1,2,3,7,8,9-HxCDD | EXP | 3.23 | 2.75 | 2.61 | 1.25 | 0.15 |
| | CTL | 0.94 | | 3.22 | | 0.05 |
| 1,2,3,4,6,7,8-HpCDD | EXP | 18.90 | 34.47 | 10.61 | 7.27 | 0.06 |
| | CTL | 11.84 | | 29.49 | | 0.02 |
| OCDD | EXP | 43.83 | 636.29 | 24.35 | 6.29 | 0.01 |
| | CTL | 12.59 | | 44.44 | | 0.00 |
| 2,3,7,8-TCDF | EXP | 3.28 | 0.91 | 0.46 | 1.25 | 0.48 |
| | CTL | 1.25 | | 3.55 | | 0.05 |
| 1,2,3,7,8-PeCDF | EXP | | | < LOD | | |
| | CTL | | | | | |
| 2,3,4,7,8-PeCDF | EXP | 8.59 | 4.82 | 1.09 | 1.27 | 0.17 |
| | CTL | 0.92 | | 3.49 | | 0.15 |
| 1,2,3,4,7,8-HxCDF | EXP | 7.49 | 8.48 | 2.88 | 1.80 | 0.07 |
| | CTL | 1.26 | | 3.55 | | 0.06 |
| 1,2,3,6,7,8-HxCDF | EXP | 3.62 | 5.40 | 1.98 | 1.47 | 0.06 |
| | CTL | 1.16 | | 3.59 | | 0.06 |
| 1,2,3,7,8,9-HxCDF | EXP | | | < LOD | | |
| | CTL | | | | | |
| 2,3,4,6,7,8-HxCDF | EXP | 3.92 | 3.84 | 0.84 | 1.54 | 0.07 |
| | CTL | 1.20 | | 4.74 | | 0.07 |
| 1,2,3,4,6,7,8-HpCDF | EXP | 13.17 | 16.31 | 5.68 | 1.72 | 0.02 |
| | CTL | 12.95 | | 15.95 | | 0.01 |
| 1,2,3,4,7,8,9-HpCDF | EXP | | | < LOD | | |
| | CTL | | | | | |
| OCDF | EXP | 5.54 | 17.85 | 1.88 | 6.28 | 0.00 |
| | CTL | 5.10 | | 54.69 | | 0.00 |
| Sum Raw min | EXP | 136.5 | 757.5 | 57.0 | 13.6 | 2.5 |
| | CTL | 38.3 | | 144.6 | | 0.0 |
| Sum Raw max | EXP | 140.5 | 758.0 | 61.2 | 39.3 | 2.6 |
| | CTL | 56.6 | | 192.1 | | 1.3 |
| Sum TEQ min[c] | EXP | 14.00 | 13.53 | 3.00 | 0.07 | 2.53 |
| | CTL | 1.22 | | 0.48 | | 0.04 |
| Sum TEQ max[c] | EXP | 14.51 | 13.58 | 3.69 | 3.38 | 2.58 |
| | CTL | 3.00 | | 9.03 | | 1.29 |

In gray the measurement lower than the limit of detection (LOD) are reported as equal to LOD.

[a]The five depurated (EXP) ewes were formerly (until day 0) fed with a hay contaminated with PCDD/Fs, and further received a non-contaminated hay until 188 days of depuration. The four control (CTL) ewes were continuously fed with non-contaminated hay. At day 0, ewes were lactating on average at 29 days in milk, weaning / dry-off took place at depuration day 63, and thereafter ewes were non-lactating and non-gestating until the end of the depuration (day 188). Results reported are for pool of individual cerumen samples within treatment and depuration day.

[b]Upper-bound values (fix to LOD when recorded at level < LOD), unless for Sum Raw min and Sum TEQ min where LOD values are set to 0.

[c]Results normalized for TEQ are determined according to the WHO 2005 TEF values.[35]

**Table S9.** Depuration kinetic of ewe sternal subcutaneous adipose tissue concentrations in polychlorinated dibenzo-*p*-dioxins and dibenzofurans (PCDD/Fs).[a]

| PCDD/F congener (pg g$^{-1}$ lipids)[b] | Treatment | Depuration days 0 | | 32 | 60 | | 130 | 188 | | SEM | *p*-value Treatment | Day | Trt × Day |
|---|---|---|---|---|---|---|---|---|---|---|---|---|---|
| 2,3,7,8-TCDD | EXP | 2.18 a | * | 1.02 a,b | 0.62 b | * | 0.12 c | 0.19 c | | 0.178 | <0.001 | <0.001 | <0.01 |
| | CTL | 0.26 a | | | 0.11 a b | | | 0.11 b | | | | | |
| 1,2,3,7,8-PeCDD | EXP | 12.86 a | * | 7.52 b | 3.66 c | * | 0.81 d | 0.58 d | * | 0.709 | <0.001 | <0.001 | 0.01 |
| | CTL | 0.75 a | | | 0.41 b | | | 0.20 b | | | | | |
| 1,2,3,4,7,8-HxCDD | EXP | 9.71 a | * | 6.42 a | 5.38 a | * | 1.47 b | 0.86 b | * | 0.752 | <0.001 | <0.001 | 0.04 |
| | CTL | 1.00 a | | | 0.62 a,b | | | 0.36 b | | | | | |
| 1,2,3,6,7,8-HxCDD | EXP | 12.59 a | * | 7.36 a | 6.53 a | * | 1.70 b | 1.36 b | □ | 1.083 | <0.001 | <0.01 | 0.02 |
| | CTL | 0.97 | | | 0.75 | | | 0.83 | | | | | |
| 1,2,3,7,8,9-HxCDD | EXP | 4.82 a | * | 2.46 a,b | 1.18 b | * | 0.48 c | 0.38 c | | 0.600 | <0.001 | <0.001 | 0.07 |
| | CTL | 1.15 a | | | 0.14 b | | | 0.32 b | | | | | |
| 1,2,3,4,6,7,8-HpCDD | EXP | 15.65 a | * | 12.50 a,b | 9.59 b | * | 3.79 c | 2.40 c | | 1.958 | <0.001 | <0.001 | 0.05 |
| | CTL | 4.33 a | | | 1.56 b | | | 1.62 b | | | | | |
| OCDD | EXP | 35.70 a | | 7.14 b | 2.77 b | | 4.77 b | 5.19 b | | 6.938 | 0.03 | <0.001 | 0.45 |
| | CTL | 10.83 a | | | 1.48 b | | | 4.02 a,b | | | | | |
| 2,3,7,8-TCDF | EXP | | | | | | | < LOD | | | | | |
| | CTL | | | | | | | | | | | | |
| 1,2,3,7,8-PeCDF | EXP | | | | | | | < LOD | | | | | |
| | CTL | | | | | | | | | | | | |
| 2,3,4,7,8-PeCDF | EXP | 10.07 a | * | 7.26 b | 3.33 c | * | 1.04 d | 0.73 d | * | 0.704 | <0.001 | <0.001 | <0.01 |
| | CTL | 0.69 a | | | 0.46 a,b | | | 0.41 b | | | | | |
| 1,2,3,4,7,8-HxCDF | EXP | 8.70 a | * | 7.50 a | 6.45 a | * | 1.43 b | 0.85 b | * | 0.846 | <0.001 | <0.001 | 0.02 |
| | CTL | 1.12 a | | | 0.68 a,b | | | 0.41 b | | | | | |
| 1,2,3,6,7,8-HxCDF | EXP | 6.20 a | * | 4.33 a | 3.37 a | * | 0.76 b | 0.40 b | * | 0.395 | <0.001 | <0.001 | 0.04 |
| | CTL | 0.96 a | | | 0.37 a,b | | | 0.17 b | | | | | |
| 1,2,3,7,8,9-HxCDF | EXP | | | | | | | < LOD | | | | | |
| | CTL | | | | | | | | | | | | |
| 2,3,4,6,7,8-HxCDF | EXP | 4.86 a | * | 3.61 a,b | 1.88 b | * | 0.61 c | 0.33 c | * | 0.388 | <0.001 | <0.001 | 0.14 |
| | CTL | 1.00 a | | | 0.20 b | | | 0.09 b | | | | | |
| 1,2,3,4,6,7,8-HpCDF | EXP | 7.88 a | * | 6.50 a | 5.87 a | * | 2.19 b | 2.42 b | | 0.974 | <0.001 | <0.01 | 0.06 |
| | CTL | 2.29 | | | 0.73 | | | 1.42 | | | | | |
| 1,2,3,4,7,8,9-HpCDF | EXP | 0.87 a | | 2.58 a | 0.79 a | * | 0.16 b | 0.12 b | | 0.560 | 0.22 | <0.001 | <0.01 |
| | CTL | 1.44 a | | | 0.23 b | | | 0.13 b | | | | | |
| OCDF | EXP | 2.66 a | | 3.96 a | 1.15 a | * | 0.61 b | 1.29 a | * | 0.979 | 0.04 | <0.001 | 0.12 |
| | CTL | 2.34 a | | | 0.28 b | | | 1.12 a | | | | | |
| Sum Raw min | EXP | 133.9 a | * | 61.2 b | 49.3 b | * | 19.8 c | 15.9 c | | 12.19 | <0.001 | <0.001 | 0.01 |
| | CTL | 18.0 | | | 7.0 | | | 11.1 | | | | | |
| Sum Raw max | EXP | 136.9 a | * | 84.6 a,b | 54.7 b | * | 20.3 c | 18.9 c | | 11.32 | <0.001 | <0.001 | <0.01 |
| | CTL | 31.7 a | | | 8.5 b | | | 12.6 b | | | | | |
| Sum TEQ min[c] | EXP | 22.89 a | * | 13.00 a, b | 7.47 b | * | 1.85 c | 1.63 c | | 1.364 | <0.001 | <0.001 | <0.01 |
| | CTL | 0.82 | | | 0.79 | | | 0.76 | | | | | |
| Sum TEQ max[c] | EXP | 23.18 a | * | 14.48 a | 8.09 b | * | 1.95 c | 1.63 c | * | 1.431 | <0.01 | <0.01 | 0.01 |
| | CTL | 2.15 a | | | 0.99 b | | | 0.82 b | | | | | |

a-d within row, least-square means with different letters differ at $p \leq 0.05$.
* At the specific depuration day, EXP and CTL least-square means differ at $p \leq 0.05$.
□ At the specific depuration day, EXP and CTL least-square means tend to differ at $p \leq 0.10$.
In gray the measurement lower than the limit of detection (LOD) are reported as equal to LOD.
[a]The five depurated (EXP) ewes were formerly (until day 0) fed with a hay contaminated with PCDD/Fs, and further received a non-contaminated hay until 188 days of depuration. The four control (CTL) ewes were continuously fed with non-contaminated hay. At day 0, ewes were lactating on average at 29 days in milk, weaning / dry-off took place at depuration day 63, and thereafter ewes were non-lactating and non-gestating until the end of the depuration (day 188). Results reported are for pool of individual cerumen samples within treatment and depuration day.
[b]Upper-bound values (fix to LOD when recorded at level <LOD), unless for Sum Raw min and Sum TEQ min where LOD values are set to 0.
[c]Results normalized for TEQ are determined according to the WHO 2005 TEF values.[35]

**Table S10.** Tissue distribution of polychlorinated dibenzo-*p*-dioxins and dibenzofurans (PCDD/Fs) in ewes at slaughter.[a]

| | | Tissue | | | | | | *p*-value | | |
|---|---|---|---|---|---|---|---|---|---|---|
| Item | Trt | Empty body | Sternal adipose tissue | LT muscle | Liver | Blood serum | SEM | Trt | Tissue | Trt × Tissue |
| Lipid content (%) | EXP | 24.4 b | 83.6 a | 4.5 c | 5.9 c | 0.17 d | 2.29 | 0.97 | <0.001 | 0.92 |
| | CTL | 22.5 b | 83.9 a | 6.7 c | 5.8 c | 0.16 d | | | | |
| PCDD/F congener (pg g$^{-1}$ lipids)[b] | | | | | | | | | | |
| 2,3,7,8-TCDD | EXP | 0.09 c,d | 0.19 b | 0.07 d | 0.14 b c * | 0.49 a | 0.057 | 0.01 | <0.001 | 0.88 |
| | CTL | 0.06 b,c | 0.11 a,b | 0.04 c | 0.04 c | 0.24 a | | | | |
| 1,2,3,7,8-PeCDD | EXP | 0.50 b □ | 0.58 b * | 0.38 b * | 2.01 a * | 0.53 b | 0.302 | <0.01 | <0.001 | 0.30 |
| | CTL | 0.21 | 0.20 a,b | 0.10 b | 0.41 a | 0.38 a | | | | |
| 1,2,3,4,7,8-HxCDD | EXP | 0.76 b * | 0.86 b * | 0.66 b * | 5.28 a * | 0.64 b | 0.500 | <0.01 | <0.001 | 0.13 |
| | CTL | 0.35 b | 0.36 b | 0.17 c | 1.12 a | 0.43 b | | | | |
| 1,2,3,6,7,8-HxCDD | EXP | 0.64 c | 1.36 a,b | 0.68 b,c □ | 3.36 a * | 3.53 a * | 0.672 | 0.02 | <0.01 | 0.53 |
| | CTL | 0.42 | 0.83 a,b | 0.23 b | 0.97 a | 0.43 a b | | | | |
| 1,2,3,7,8,9-HxCDD | EXP | 0.08 c | 0.38 b | 0.12 c | 0.84 a,b * | 1.51 a | 0.238 | 0.38 | <0.001 | 0.05 |
| | CTL | 0.20 b | 0.32 a,b | 0.19 b | 0.30 a,b | 0.52 a | | | | |
| 1,2,3,4,6,7,8-HpCDD | EXP | 1.81 c | 2.40 c □ | 1.77 c | 13.14 a * | 6.41 b * | 1.086 | <0.01 | <0.001 | 0.56 |
| | CTL | 1.52 b c | 1.62 b c | 1.28 c | 6.36 a | 2.67 b | | | | |
| OCDD | EXP | 1.59 c | 5.19 b | 3.91 b | 6.25 b | 18.06 a * | 2.245 | 0.20 | <0.001 | <0.01 |
| | CTL | 2.37 c | 4.02 b,c | 2.77 c | 9.05 a | 6.46 b | | | | |
| 2,3,7,8-TCDF | EXP | 1.31 a | 1.65 a | 0.07 b | 0.12 b | 4.77 a * | 0.770 | 0.03 | <0.001 | 0.57 |
| | CTL | 0.85 a | 1.19 a | 0.09 b | 0.11 b | 0.52 a | | | | |
| 1,2,3,7,8PeCDF | EXP | | | | < LOD | | | | | |
| | CTL | | | | | | | | | |
| 2,3,4,7,8-PeCDF | EXP | 0.56 b,c * | 0.73 b * | 0.47 c * | 14.24 a * | 0.58 b,c | 1.481 | <0.01 | <0.001 | 0.08 |
| | CTL | 0.25 b,c | 0.41 b | 0.18 c | 3.15 a | 0.50 b | | | | |
| 1,2,3,4,7,8-HxCDF | EXP | 0.81 b * | 0.85 b * | 0.63 b * | 17.68 a * | 0.70 b | 1.714 | <0.01 | <0.001 | 0.08 |
| | CTL | 0.34 b,c | 0.41 b,c | 0.21 c | 3.73 a | 0.59 b | | | | |
| 1,2,3,6,7,8-HxCDF | EXP | 0.37 b * | 0.40 b * | 0.32 b * | 12.28 a * | 0.59 b | 1.265 | <0.001 | <0.001 | <0.01 |
| | CTL | 0.17 c | 0.17 c | 0.15 c | 2.20 a | 0.60 b | | | | |
| 1,2,3,7,8,9-HxCDF | EXP | | | | < LOD | | | | | |
| | CTL | | | | | | | | | |
| 2,3,4,6,7,8-HxCDF | EXP | 0.29 c □ | 0.33 c * | 0.19 d | 10.29 a * | 0.71 b | 0.954 | <0.01 | <0.001 | 0.07 |
| | CTL | 0.17 c | 0.09 c | 0.11 c | 2.03 a | 0.68 b | | | | |
| 1,2,3,4,6,7,8-HpCDF | EXP | 0.98 c | 2.42 b * | 0.96 c | 38.15 a * | 1.82 b | 3.704 | <0.01 | <0.001 | 0.06 |
| | CTL | 0.71 b | 1.42 b | 0.62 b | 7.35 a | 1.26 b | | | | |
| 1,2,3,4,7,8,9-HpCDF | EXP | 0.09 c * | 0.12 c | 0.13 c | 1.81 a * | 0.51 b * | 0.168 | 0.67 | <0.001 | <0.01 |
| | CTL | 0.25 b | 0.13 b,c | 0.12 c | 0.51 a | 0.91 a | | | | |
| OCDF | EXP | 0.33 b | 1.29 a | 0.38 b | 2.20 a □ | 2.17 a | 0.343 | 0.71 | <0.001 | 0.28 |
| | CTL | 0.59 b,c | 1.12 a,b | 0.52 c | 1.04 a,b | 2.00 a | | | | |
| Sum Raw min | EXP | 10.0 c | 15.8 c □ | 10.4 c | 127.7 a * | 39.6 b * | 11.32 | <0.01 | <0.001 | 0.17 |
| | CTL | 7.0 b | 11.1 b | 6.3 b | 38.3 a | 10.3 b | | | | |
| Sum Raw max | EXP | 10.3 d | 18.9 c □ | 10.9 d □ | 127.9 a * | 43.6 b * | 11.11 | <0.01 | <0.001 | 0.11 |
| | CTL | 8.7 c | 12.6 b,c | 6.9 c | 38.5 a | 19.9 b | | | | |
| Sum TEQ min[c] | EXP | 1.20 b | 1.63 b | 0.81 b □ | 11.91 a * | 2.05 b * | 1.241 | <0.01 | <0.001 | 0.24 |
| | CTL | 0.46 b | 0.76 b | 0.24 b | 2.54 a | 0.02 c | | | | |
| Sum TEQ max[c] | EXP | 1.21 c,d □ | 1.63 b,c * | 0.89 d * | 11.94 a | 2.58 b □ | 1.218 | <0.001 | <0.001 | 0.30 |
| | CTL | 0.63 b,c | 0.82 b | 0.34 c | 2.59 a | 1.28 b | | | | |

a-d within row, least-square means with different letters differ at $p \leq 0.05$.
* At the specific depuration day, EXP and CTL least-square means differ at $p \leq 0.05$.
□ At the specific depuration day, EXP and CTL least-square means tend to differ at $p \leq 0.10$.
In gray the measurement lower than the limit of detection (LOD) are reported as equal to LOD.
Trt: treatment, LT muscle: *Longissimus thoracis* muscle.
[a]The five depurated (EXP) ewes were formerly (until day 0) fed with a hay contaminated with PCDD/Fs, and further received a non-contaminated hay until 188 days of depuration. The four control (CTL) ewes were continuously fed with non-contaminated hay. At day 0, ewes were lactating on average at 29 days in milk, weaning / dry-off took place at depuration day 63, and thereafter ewes were non-lactating and non-gestating until the end of the depuration (day 188).
[b]Upper-bound values (fix to LOD when recorded at level <LOD), unless for Sum Raw min and Sum TEQ min where LOD values are set to 0.
[c]Results normalized for TEQ are determined according to the WHO 2005 TEF values.[35]

**Table S11.** Body chemical composition and polychlorinated dibenzo-*p*-dioxin and dibenzofuran (PCDD/F) burdens of ewes at slaughter.[a]

| Item | Treatment | | SEM | *p*-value |
|---|---|---|---|---|
| | EXP | CTL | | |
| Body weight (kg) | 56.9 | 62.2 | 3.31 | 0.30 |
| Empty body weight (kg) | 46.0 | 48.3 | 23.13 | 0.89 |
| Hot carcass weight (kg) | 25.0 | 27.0 | 1.58 | 0.42 |
| Perirenal adipose tissue weight (kg) | 1.201 | 0.963 | 0.3146 | 0.61 |
| Empty body chemical component masses (kg) | | | | |
| Water | 26.7 | 28.7 | 1.30 | 0.31 |
| Lipids | 11.5 | 11.1 | 2.21 | 0.92 |
| Proteins | 7.2 | 7.9 | 0.32 | 0.17 |
| Minerals | 2.0 | 2.3 | 0.09 | 0.06 |
| Empty body chemical component proportions (%) | | | | |
| Water | 58.3 | 59.8 | 2.33 | 0.68 |
| Lipids | 24.4 | 22.5 | 3.36 | 0.71 |
| Proteins | 15.8 | 16.4 | 0.43 | 0.33 |
| Minerals | 4.4 | 4.7 | 0.28 | 0.46 |
| Empty body PCDD/F burdens (ng)[b] | | | | |
| 2,3,7,8-TCDD | 1.00 | 0.57 | 0.172 | 0.12 |
| 1,2,3,7,8-PeCDD | 5.63 | 2.50 | 1.517 | 0.19 |
| 1,2,3,4,7,8-HxCDD | 8.61 | 4.32 | 1.680 | 0.12 |
| 1,2,3,6,7,8-HxCDD | 7.15 | 5.21 | 1.856 | 0.49 |
| 1,2,3,7,8,9-HxCDD | < LOD | | | |
| 1,2,3,4,6,7,8-HpCDD | 20.84 | 18.17 | 5.520 | 0.74 |
| OCDD | 17.54 | 25.87 | 4.138 | 0.33 |
| 2,3,7,8-TCDF | 14.42 | 7.43 | 1.887 | 0.04 |
| 1,2,3,7,8-PeCDF | < LOD | | | |
| 2,3,4,7,8-PeCDF | 6.56 | 2.94 | 1.576 | 0.15 |
| 1,2,3,4,7,8-HxCDF | 9.25 | 4.17 | 1.755 | 0.08 |
| 1,2,3,6,7,8-HxCDF | 4.36 | 1.87 | 0.907 | 0.10 |
| 1,2,3,7,8,9-HxCDF | < LOD | | | |
| 2,3,4,6,7,8-HxCDF | 3.34 | 1.70 | 0.702 | 0.15 |
| 1,2,3,4,6,7,8-HpCDF | 10.89 | 8.21 | 2.438 | 0.47 |
| 1,2,3,4,7,8,9-HpCDF | < LOD | | | |
| OCDF | 3.88 | 5.74 | 1.308 | 0.35 |
| Sum Raw min | 113.5 | 80.4 | 22.22 | 0.33 |
| Sum Raw max | 116.6 | 96.7 | 24.17 | 0.58 |
| Sum TEQ min[c] | 13.67 | 5.85 | 2.860 | 0.10 |
| Sum TEQ max[c] | 13.82 | 7.19 | 2.889 | 0.15 |

In gray the measurement lower than the limit of detection (LOD) are reported as equal to LOD.
[a]The five depurated (EXP) ewes were formerly (until day 0) fed with a hay contaminated with PCDD/Fs, and further received a non-contaminated hay until 188 days of depuration. The four control (CTL) ewes were continuously fed with non-contaminated hay. At day 0, ewes were lactating on average at 29 days in milk, weaning / dry-off took place at depuration day 63, and thereafter ewes were non-lactating and non-gestating until the end of the depuration (day 188).
[b]Upper-bound values (fix to LOD when recorded at level <LOD), unless for Sum Raw min and Sum TEQ min where LOD values are set to 0.
[c]Results normalized for TEQ are determined according to the WHO 2005 TEF values.[35]
